# Insights into Liposomal and Gel-Based Formulations for Dermatological Treatments

**DOI:** 10.3390/gels11040245

**Published:** 2025-03-26

**Authors:** Giovanni Strazzabosco, Alessia Liboni, Giulia Pezzi, Andrea Alogna, Daria Bortolotti

**Affiliations:** 1 Department of Chemical, Pharmaceutical and Agricultural Sciences, University of Ferrara, 44121 Ferrara, Italy; giovanni.strazzabosco@unife.it (G.S.); alessia.liboni@unife.it (A.L.); 2 Department of Environmental and Prevention Sciences, University of Ferrara, 44121 Ferrara, Italy; giulia.pezzi@unife.it (G.P.); daria.bortolotti@unife.it (D.B.)

**Keywords:** liposomal formulation, gel-based formulations, dermatological treatments, drug delivery systems

## Abstract

Dermatological diseases pose a significant challenge due to their chronic nature, complex pathophysiology, and the need for effective, patient-friendly treatments. **Recent** advancements in liposomal and gel-based formulations have played a crucial role in improving drug delivery, therapeutic efficacy, and patient compliance. Liposomal formulations have garnered considerable attention in dermatology due to their ability to encapsulate both hydrophilic and lipophilic compounds, enabling controlled drug release and enhanced skin penetration. However, challenges such as formulation complexity, stability issues, and regulatory constraints remain. Similarly, gel-based formulations are widely used due to their ease of application, biocompatibility, and ability to retain active ingredients. However, they also face limitations, including restricted penetration depth, susceptibility to microbial contamination, and challenges in achieving sustained drug release. The integration of liposomal and gel-based technologies offers a promising strategy to overcome current challenges and optimize dermatological drug delivery. This review explores both well-established therapies and recent innovations, offering a comprehensive overview of their applications in the treatment of prevalent dermatological conditions. Ultimately, continued research is essential to refine these formulations, expanding their clinical utility and enhancing therapeutic effectiveness in dermatology.

## 1. Introduction

Human skin is the largest organ of the human body and is recognized as a complex and dynamic structure with multiple essential functions. It accounts for approximately 15% of the body’s weight and spans an area of around 2 m^2^ in a 70 kg person. The thickness of the skin varies between 1.5 and 5.0 mm [1]. It serves as a protective barrier, shielding the body from pathogens, harmful substances, injuries, and dehydration. Additionally, the skin contains receptors that facilitate the perception of touch, pressure, pain, and temperature [2].

Moreover, the skin plays a vital role in the thermoregulation process by modulating body temperature using different mechanisms such as sweating and blood vessel dilation/constriction [3]. When exposed to ultraviolet (UV) radiation, the skin produces vitamin D, which is essential for nutrient metabolism. Furthermore, it produces antimicrobial peptides, cytokines, and growth factors that intervene in the inflammatory process, helping the immune system while aiding in the tissue repair mechanism [4]. The appearance of skin is influenced by many factors. These include the amount of oxygen present, the concentration of eumelanin and pheomelanin, the distribution and size of hairs and glands, and the aging process [5]. This organ is composed of three main layers with different functions and characteristics: epidermis, dermis, and hypodermis.

### 1.1. Skin Disorders

Skin disorders encompass a wide range of conditions that affect skin, hair, and nails, varying significantly in severity, clinical presentation, and underlying etiologies [6]. These dermatological conditions include inflammatory diseases, such as psoriasis and atopic dermatitis [7], skin cancers [8], complications related to wound care [9], and infection management [10]. Collectively, skin disorders pose a considerable challenge to global health systems, accounting for approximately 1.79% of the overall disease burden [11].

The treatment of these conditions often requires diverse therapeutic approaches; however, even short-term therapies can lead to adverse effects (Table 1). Inflammatory skin disorders are prevalent and demand effective management strategies. Current treatments typically involve topical and systemic therapies (e.g., corticosteroids), which provide immediate relief but carry significant risks [12]. Atopic dermatitis (AD) serves as a prime example of the complexity of managing dermatological conditions. Characterized by eczema, severe itching, and a compromised skin barrier, atopic dermatitis is a multifactorial skin condition triggered by immune dysregulation, microbial imbalance, and environmental factors, including sun exposure, stress, and hygiene practices [13,14]. Despite the widespread use of topical corticosteroids as a primary treatment, there are concerns regarding adverse effects, such as atrophy, striae, rosacea, perioral dermatitis, acneiform eruption, and purpura [15]. While cosmetic products like moisturizers and emollients are commonly recommended to improve skin hydration and repair barrier function, their clinical efficacy remains uncertain [16]. Consequently, there is a growing demand for targeted therapies that minimize side effects while maximizing therapeutic efficacy and duration.

Rosacea is another chronic inflammatory disorder that currently lacks a definitive cure. Its development is influenced by both genetic predispositions and environmental factors. Clinically, rosacea is characterized by persistent facial redness, telangiectasia, and inflammatory papules or pustules that may occur individually or together [17]. Treatment primarily consists of topical and systemic agents that target adrenergic pathways, such as oxymetazoline, which has been shown to reduce erythema [18]. Psoriasis exemplifies the multifaceted challenges presented by chronic inflammatory skin diseases [19]. This immune-mediated disorder is marked by excessive keratinocyte proliferation, leading to erythema, scaling, and pruritus [20]. Management strategies vary depending on disease severity; first-line treatments typically include topical corticosteroids and vitamin D analogs [21,22]. The inflammatory skin diseases discussed share common treatment limitations, including insufficient long-term efficacy and the emergence of adverse effects, underscoring the need for alternative therapeutic approaches. Significant progress has been made with the introduction and commercialization of biologics and biosimilar drugs, which target key components of the inflammatory pathways associated with skin inflammation, such as IL-17 and IL-23. These biologics have shown considerable effectiveness in achieving sustained disease control [23]. However, despite their reduced incidence of side effects, their high costs and the requirement for intravenous or localized injection routes often prevent many patients from accessing these beneficial treatments [24].

### 1.2. Wound Care and Skin Cancer

Another challenge in dermatology is the management of wound healing. Skin wounds pose significant challenges to public health and the economy, often leading to complications that can severely impact patients’ quality of life [25]. Additionally, they can adversely affect mental well-being, potentially leading to psychological conditions such as depression, anxiety, and social isolation [26]. Various factors contribute to wound formation, including surgical procedures, injuries from external causes like burns or cuts, and medical conditions like diabetes [27]. Wound healing begins immediately after an injury involving, for example, the epidermal layer. This process involves tissue regeneration as the body responds to the damaged cells caused by injuries [28]. Healing is a time-consuming endeavor that encompasses several overlapping phases: hemostasis, inflammation, proliferation, and remodeling [29]. However, if this process is disrupted, tissue regeneration is hindered, potentially leading to abnormal scars or prolonged inflammation that delays recovery. Chronic wounds are a primary example of this complication [30]. Moreover, these latter conditions provide an ideal environment for bacterial infiltration and growth, increasing the risk of persistent infections [31]. Various treatment options are available based on wound type, including infection swabbing, wound bed cleaning, transplantation, cell therapy, wound dressings, and advanced instrumental techniques [32]. Poor wound healing, especially in chronic lesions, is associated with increased mortality rates and significant healthcare costs [33].

A critical dermatological concern is skin cancer. There has been a notable global rise in skin cancer cases, which is recognized as the most prevalent type of cancer in humans. Most cases are non-melanomatous (NMSCs), including basal cell carcinoma (BCC) and squamous cell carcinoma (SCC) [34]. BCC accounts for approximately 70–75% of NMSC cases, while SCC makes up about 20–25% [35]. These cancers generally arise in the upper layers of the skin, particularly the epidermis, and are strongly associated with prolonged exposure to ultraviolet (UV) radiation from sunlight or tanning beds [36]. Although cutaneous melanoma constitutes only about 1.7% of all malignant skin cancers, it is responsible for the majority of deaths related to skin cancer due to its aggressive nature despite its lower incidence compared to non-melanoma types [37]. Melanoma originates from melanocytes located in the basal layer of the epidermis that produce the UV-absorbing pigment melanin [38]. UV exposure can lead to the accumulation of genetic mutations, activating oncogenes while inactivating tumor suppressor genes and impairing DNA repair mechanisms [39]. This cascade results in uncontrolled melanocyte proliferation, leading to melanoma. Early-stage melanoma is often treatable through surgery, with high survival rates; however, once metastasis occurs, survival rates decline significantly [40]. Thus, early and accurate diagnosis is crucial for improving patient outcomes [41].

### 1.3. Skin Infections and Inflammation

Skin inflammation and damage can also be caused by abnormal colonization by different types of microorganisms. Normally, bacteria, fungi, and viruses naturally coexist with human hosts [42], and the maintenance of a balanced microbiome is crucial for skin health. When this balance is disrupted, often due to factors such as poor hygiene, environmental changes, or underlying health conditions, pathogenic microorganisms can proliferate, leading to infections and subsequent inflammation [43]. For instance, an overgrowth of certain bacteria like Staphylococcus aureus can trigger conditions such as eczema or acne [44,45], while fungal infections may arise from an imbalance involving yeasts like Malassezia [46]. Additionally, the skin’s microbiome plays a significant role in immune regulation and barrier function, and any dysbiosis can compromise these protective mechanisms, increasing susceptibility to infections and skin diseases.

Susceptibility to cutaneous infections is significantly elevated when the skin barrier is compromised [47]. Epidermal disruptions, such as lacerations or abrasions, diminish the host’s innate defense mechanisms against microbial invasion. Furthermore, dermatological conditions characterized by barrier dysfunction and immunological aberrations, including atopic dermatitis and psoriasis, are associated with an increased propensity for bacterial colonization and subsequent infection [48]. Treatment for this skin condition can vary widely, from conservative management to the use of emollients and antibiotics; in severe cases, hospitalization may be necessary [49]. It is crucial to closely monitor patients with bacterial infections, tracking the spread of inflammation and documenting changes on a body map.

In contrast, viral skin infections often present with systemic symptoms and can be highly contagious through direct contact with infected individuals or contaminated surfaces [50]. The most prevalent viral infection affecting the skin is caused by Herpes Simplex virus (HSV) [51], leading to conditions such as cold sores or genital herpes [52]. Other viral infections include Varicella-zoster virus (VZV), responsible for chickenpox and shingles [53], as well as Human Papillomavirus (HPV), which can cause warts or certain types of skin cancer [54].

Individuals with compromised immune systems are particularly vulnerable to more aggressive forms of these infections. Management typically involves symptomatic treatment; however, antiviral medications may be indicated in several cases [55]. Fungal infections can also lead to superficial skin issues but are often diagnosed and treated only when patients seek healthcare for other concerns [56]. These infections are primarily caused by two groups of fungi: dermatophytes and yeasts (candidiasis) [57]. Areas prone to fungal infections include interdigital spaces (such as between the toes) and skin folds [58]. Fungal infections are commonly observed in patients who have recently taken antibiotics, which can disrupt the skin microbiome [59]. Additionally, individuals with underlying medical conditions [60], as well as pregnant [61] and obese individuals [62], are at increased risk. Localized fungal infections are typically treated with topical antifungals, such as creams or powders, for one to four weeks. If the affected skin is particularly sore, red, and painful, topical steroids may be prescribed alongside antifungal treatment to provide additional relief [63].

### 1.4. Targeted Drug Delivery in Dermatology

Beyond the specific conditions discussed above, primary objectives in dermatological disease management remain focused on reducing inflammation, alleviating symptoms, and preventing exacerbations. Effective management strategies are essential for improving patient outcomes and enhancing quality of life. Current therapeutic strategies often influence the effectiveness of drug penetration into the skin, highlighting the critical need for innovative drug delivery systems in dermatology. Targeted drug delivery has emerged as a transformative approach designed to enhance localized drug action while minimizing systemic exposure and allowing sustained release of active ingredients [64].

Recent studies highlight the significant impact of liposomal formulations on drug penetration and flux modification in both in vitro and in vivo models. Due to their amphiphilic nature and ability to encapsulate both hydrophilic and lipophilic compounds, they enhance drug permeability through the stratum corneum, thereby increasing bioavailability at the target site. For example, a comparative study using Franz diffusion cells showed that liposomal formulations improved skin penetration by approximately 3–5 times compared to conventional creams or gels, with a two-fold increase in drug retention within the epidermis [65]. Similarly, in vivo studies on animal models reported that liposome-encapsulated drugs exhibited a 40–60% increase in dermal drug concentration compared to non-liposomal formulations, leading to prolonged therapeutic effects and reduced systemic absorption [66]. Moreover, deformable liposomes, such as transferosomes and ethosomes [67], have been found to enhance transdermal drug flux by up to 10-fold [68], making them particularly effective for delivering macromolecules and hydrophilic drugs across the skin barrier. These advancements highlight the potential of liposomal formulations in optimizing dermatological drug delivery by increasing drug permeation, prolonging retention, and reducing side effects.

Among the most promising innovations, liposomal and gel-based formulations have been extensively studied and continuously improved to increase treatment efficacy. Liposomes, characterized by their biocompatible lipid bilayer structure, can encapsulate both hydrophilic and hydrophobic drugs, enhancing stability and promoting deeper skin penetration [69]. Meanwhile, hydrogels provide a versatile platform for incorporating active compounds, offering benefits such as enhanced hydration, prolonged release, and improved patient comfort.

**Table 1:** Division of topical and systemic therapies for the most well-known skin pathologies, along with high incidence side effects of such therapies.

Therapy	Side Effects	Skin Pathology
**Topical Therapies**		
*Corticosteroids* (e.g., hydrocortisone and betamethasone)	Skin thinning, skin atrophy, telangiectasia, irritation, folliculitis, and striae	Psoriasis [19,21] and atopic dermatitis (AD) [13,14]
*Phototherapy* (e.g., narrow-band UVB, Psoralen + UV-A − PUVA) and photodynamic therapy (PDT)	Nausea, skin burning, and increased skin cancer risk	Atopic dermatitis (AD) [13], psoriasis [19,21], and skin cancer [8]
*Antibiotics* (e.g., clindamycin, tetracycline, macrolides, and isotretinoin)	Antibiotics resistance, gastrointestinal issues, and nausea	Rosacea [17], acne [44], skin infections [43], and skin wounds [9,25]
*Chemotherapy* (5-fluorouracil and imiquimod)	Irritation, redness, pigmentation changes, and skin ulceration	Squamous cells carcinoma, basal cell carcinoma [34]
**Systemic Therapies**		
*Immunomodulatory* (e.g., cyclosporin, methotrexate, sulfasalazine, and cemiplimab)	Increased infection risk, renal impairment, hypertension, hypersensitivity reaction, and nausea	Psoriasis [19,21], atopic dermatitis (AD) [13], and skin cancer [8]
*Chemotherapy* (dacarbazine, paclitaxel, and cisplatin)	Nausea, skin rash, pruritus hyperpigmentation, hypersensitivity, and hair loss	Melanoma [38], squamous cells carcinoma, and basal cell carcinoma [34]
*JAK inhibitors* (e.g., abrocitinib, upadacitinib, baricitinib, deuruxolitinib, vemurafenib, and dabrafenib)	Nausea, headache, and increased risk of infections	Atopic dermatitis (AD) [13] and non-melanoma and melanoma skin cancer [8]
*TNF-α inhibitors* (e.g., adalimumab, infliximab, and remicade)	Injection site reactions and increased infection risk	Psoriasis [19,21] and acne [45]
*IL-17 inhibitors* (e.g., secukinumab, bimekizumab, and brodalumab)	Diarrhea, injection site reactions, and risk of infections	Psoriasis [19,21]

When combined with nanocarrier technologies, these systems can further enhance drug solubility and facilitate targeted penetration into skin layers [70]. These advancements offer a promising strategy to overcome the limitations of conventional drug delivery systems, potentially transforming the treatment of dermatological disorders. This discussion focuses on these formulations, analyzing their composition, applications, and limitations to provide a thorough assessment of their role in managing a wide range of skin conditions.

## 2. Liposomal Formulations

Liposomal formulations are an advanced delivery system composed of spherical vesicles made up of one or more lipid bilayers that enclose an aqueous core. These vesicles form naturally through the self-assembly of phospholipids in water, resulting in a unique amphiphilic structure where the hydrophilic heads face outward and the hydrophobic tails point inward. This distinctive arrangement makes liposomes highly versatile carriers for drugs and active ingredients. Widely utilized in both pharmaceutical and cosmetic applications, liposomal formulations offer significant advantages, particularly in the treatment of dermatological disorders.

### 2.1. Structural and Functional Properties of Liposomes

Liposomes are spherical-like vesicles formed by the self-assembly of amphiphilic lipid molecules in solution, capable of encapsulating both hydrophilic and hydrophobic compounds [71]. Their primary components are glycerophospholipids, a class of amphipathic lipids that are essential for cellular membranes.

These complex molecules consist of a glycerol backbone functionalized with two fatty acid chains esterified at the sn-1 and sn-2 positions. The length and degree of saturation of these fatty acid chains significantly influence the membrane fluidity. Additionally, each glycerophospholipid contains a phosphate group (the polar head) that can further link to other molecules, such as choline, ethanolamine, serine, inositol, or glycerol. Typically, a phospholipid bilayer structure forms, resembling the double-layered membrane found in cells.

#### 2.1.1. Classification of Liposomes Based on the Number of Lipid Bilayers

Based on the number of lipid bilayers, liposomes can be classified as small unilamellar vesicles (SUVs), large unilamellar vesicles (LUVs), multilamellar vesicles (MLVs), and multivesicular vesicles (MVVs).

- *Unilamellar Vesicles*

Small unilamellar vesicles (SUVs) consist of a single lipid bilayer surrounding an aqueous core, typically ranging in size from 20 to 100 nm. They are commonly produced through sonication [72], extrusion [73], or detergent dialysis [74].

Large unilamellar vesicles (LUVs) also feature a single lipid bilayer but are larger, with sizes between 100 and 250 nm. They are often prepared using extrusion [75] or dehydration–rehydration techniques [76];

- *Multivesicular and Multivesicular Vesicles*

**Figure 1:**
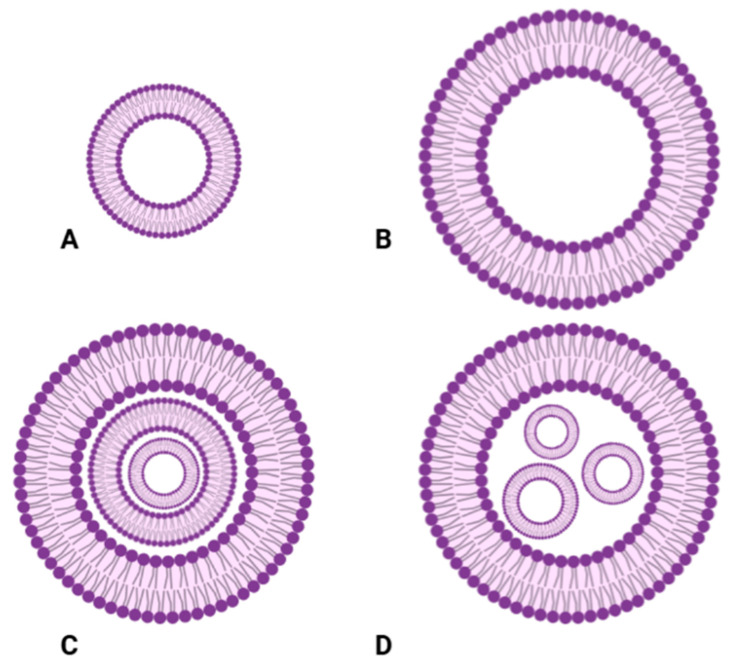
(**A**) SUVs (small unilamellar vesicles), (**B**) LUVs (large unilamellar vesicles), (**C**) MLVs (multilamellar vesicles), and (**D**) MVVs (multivesicular vesicles). Figure generated with biorender.

Multilamellar vesicles (MLVs) contain multiple concentric phospholipid bilayers, with sizes ranging from 1 to 5 µm. They can be generated through thin-film hydration [77] or reverse-phase evaporation [78]. Multivesicular vesicles (MVVs), in contrast to MLVs, encapsulate multiple unilamellar vesicles within a larger liposomal structure. Their preparation methods include cochleate cylinder formation, interdigitated bilayer techniques, bulk processes with or without double emulsion, and microfluidic approaches with or without emulsions (Table 2, Figure 1) [79].

#### 2.1.2. Structural Variations on Liposome Functionality

These structural variations influence encapsulation efficiency, drug release profiles, and biological behavior [80].

Furthermore, liposomes can be categorized not only by their lamellar composition but also by their size (usually 50–500 nm in diameter [69]), lipid bilayer rigidity, fluidity, electrical charge, lipid organization, and surface modifications (Table 3). Each of these factors plays a crucial role in altering drug delivery efficiency, release mechanisms, and liposome interactions with target tissues.

The surface charge of liposomes is determined by the chemical composition of their phospholipid head groups, which influences their interactions with biological membranes and other charged molecules [81]. For example, positively charged (cationic) liposomes tend to interact more effectively with the negatively charged surfaces of cellular membranes, enhancing cellular uptake [82].

The permeability of liposomes is influenced by both the composition of their membranes and the co-formulating agents used to achieve specific properties and functionalities. Cholesterol enhances membrane integrity by stabilizing lipid packing, reducing bilayer permeability, and controlling fluidity. This stabilization minimizes the leakage of encapsulated substances, ensuring a more controlled and sustained release [83].

#### 2.1.3. Functional Modifications of Liposomes

Further modifications can be made during the production phase of liposomes, resulting in functional derivatives that differ from the fundamental structure of liposomes, overcoming some of the limitations of this traditional nanocarrier. Transferosomes are one example of “modified” liposomes that exhibit the highest deformability among nanocarrier formulations [84], primarily due to the incorporation of surfactants such as sodium cholate, Tweens, Spans, and stearylamine [85]. This enhanced deformability significantly increases their ability to penetrate the skin; indeed, an increased delivery of diverse drugs, such as insulin and antifungal molecules, has been shown [86].

Another example of functional modification is Ethosomes. These are similar to liposomes, but their production involves a higher concentration of ethanol (e.g., 50%), which acts as an enhancer of elasticity and deformability. Typically, these nanocarriers also contain soy phosphatidylcholine (SPC), a naturally derived phospholipid that increases the entrapment efficiency of drugs within Ethosomes [87].

Additionally, the choice of lipids in formulations influences how liposomes respond to environmental factors like temperature, as well as other essential aspects such as stability and drug release characteristics. Natural lipids are frequently used for liposome production, and they are typically derived from egg yolk [88] or soybean [89]. These lipids are characterized by the presence of unsaturated bonds along the hydrocarbon chain (e.g., oleic, linoleic, and linolenic acids, each with 1, 2, and 3 unsaturated bonds, respectively), and due to this characteristic, they are considered less stable than synthetic lipids. However, this “instability” can be exploited to improve drug release profiles, as demonstrated by Luo D. et al., who proved light-triggered drug release after the oxidation of DOPC (1,2-dioleoyl-sn-glycero-3-phosphocholine) and cholesterol [90].

On the other hand, synthetic lipids lack unsaturated bonds and often feature further modifications to the polar head group. They can be obtained from pure synthesis (starting from mannitol or glycerophosphocholine (GPC) [91] or from stearic acid and palmitic fatty acid [69]). These phospholipids are widely used to optimize the drug-targeting capacity of liposomes and, in some cases, act as active pro-drugs [92].

Moreover, the temperature-related behavior of the bilayer varies depending on the type of lipids it comprises. Specifically, the phase transition temperature (the point at which the bilayer shifts from a gel phase to a liquid–crystalline phase [93]) increases with a higher concentration of saturated fatty acids, while it decreases with the presence of unsaturated fatty acids. Additionally, cholesterol plays a significant role in modulating the phase transition behavior of the liposomal bilayer [94]. By understanding these factors, researchers can fine-tune the encapsulation efficiency, effectiveness, and targeted activity of these nanocarriers [95].

#### 2.1.4. Applications of Liposomes in Target Drug Delivery

The structural similarity of liposomes to biological membranes facilitates efficient cell interactions and uptake, enabling effective drug delivery [96]. To increase the specificity of this interaction, distinct ligands like antibodies [97], fusogenic agents (e.g., DOPE) [98], proteins, or functionalized polyethylene glycol (PEG) can be attached to the nanocarrier surface, allowing a selective delivery, prolonged circulating time and reduced immune system clearance [99].

Their small size and amphiphilic nature enable passive diffusion across biological membranes. Additionally, stimuli-responsive liposomes release their cargo under specific physiological conditions. For example, pH-sensitive liposomes destabilize in acidic environments, triggering localized drug release in tumors or inflamed tissues [100,101]. Similarly, thermosensitive liposomes, which are positively charged liposomes, respond to elevated temperatures, releasing their encapsulated compounds upon hyperthermic stimuli [102].

Cationic liposomes, formed using cationic lipids (e.g., DOTAP, DODAC), enhance cellular uptake and transfection efficiency [103]. Furthermore, surface modification with specific ligands enables active targeting, directing the drug to the intended site while minimizing systemic exposure and potential side effects [104].

In addition, liposomes have found utility in other fields. They are widely used in vaccine delivery, where they act as adjuvants or carriers for antigens, enhancing the immune response [105,106]. In gene therapy, liposomes serve as vectors for delivering nucleic acids like DNA or RNA into target cells [107].

**Table 3:** Classification of liposomes based on functional modifications and their preparation method.

Type	Description	Preparations Methods
*Conventional Liposomes*	Basic liposomes used for drug delivery, typically composed of natural phospholipids	Thin-film hydration [77] and sonication
*Fusogenic Liposomes*	Designed to facilitate the fusion of the liposome with cellular membranes, enhancing drug delivery	Incorporation of fusogenic agents (e.g., DOPE) [98]
*Prolonged-Circulating Liposomes*	Modified to evade the immune system and prolong circulation time in the bloodstream	PEGylation during liposome preparation [99]
*pH-sensitive Liposomes*	Designed to release their contents in response to pH changes	Incorporation of pH-sensitive lipids during formation [100]
*Thermosensitive Liposomes*	Respond to elevated temperatures, releasing their encapsulated compounds upon hyperthermic stimuli	Positively charged liposomes that enhance cellular uptake and transfection efficiency [102]
*Cationic Liposomes*	Positively charged liposomes that enhance cellular uptake and transfection efficiency	Formed using cationic lipids (e.g., DOTAP and DODAC) [103]
*Immunoliposomes*	Engineered to target specific cells or tissues by attaching antibodies or ligands to their surface	Coupling antibodies to pre-formed liposomes [105]

In summary, the structural and functional versatility of liposomes underscores their prominence in nanotechnology, offering significant potential across medicine, cosmetics, and food science. Their capacity to encapsulate diverse compounds, interact seamlessly with biological systems, and adapt dynamically to environmental conditions makes them indispensable in advancing therapeutic, cosmetic, and nutritional innovations.

Recent advancements in liposomal technology have significantly expanded their applications for delivering a wide range of bioactive compounds, including anti-inflammatory agents, antifungals, and antioxidants, thereby enhancing both stability and therapeutic outcomes for encapsulated substances [108].

In dermatology, the unique properties of liposomes make them particularly beneficial for treating various skin conditions. Their ability to enhance penetration through the skin’s stratum corneum allows increased delivery directly to the affected areas while minimizing systemic exposure and side effects. This targeted approach is crucial in optimizing treatment efficacy and improving patient outcomes in dermatological therapies.

### 2.2. Therapeutic Applications of Liposomes in Dermatology

Liposomes have become a significant advancement in dermatological therapeutics, providing innovative solutions for various skin conditions. These lipid-based vesicles can encapsulate and deliver therapeutic agents directly to specific skin layers, enhancing drug penetration and localization [109]. This targeted approach has demonstrated efficacy in the treatment of conditions such as skin inflammation [110], skin cancer [111], and skin infection [112].

By encapsulating active pharmaceutical ingredients within their lipid bilayer structures, liposomes improve drug delivery by optimizing the concentration of therapeutic agents precisely where they are needed most. This not only increases the efficacy of treatments but also minimizes systemic absorption, reducing unwanted side effects and enhancing patient safety [113]. Furthermore, liposomes exhibit remarkable adaptability to a wide range of drug molecules and possess excellent biocompatibility, reinforcing their role as a promising tool in advancing dermatological therapies [114].

#### 2.2.1. Skin Inflammation

Inflammatory skin diseases represent a serious threat to public health worldwide, with the most common conditions being psoriasis [21], atopic dermatitis [115], and alopecia areata [116]. These chronic conditions significantly impact patients’ physical well-being and quality of life due to persistent symptoms such as erythema, itching, and scaling [117]. Traditionally, the management of inflammatory skin diseases has relied on a combination of topical and systemic therapies, including corticosteroids and immunosuppressants [118]. While these treatments can provide symptomatic relief, they frequently fail to address the underlying causes of inflammation and may lead to unsatisfactory outcomes. Moreover, prolonged use of these therapies is associated with significant adverse effects, such as skin thinning, systemic toxicity, and increased susceptibility to infections (Table 1) [119]. This underscores the need for novel therapeutic strategies that are both effective and safer for patients.

Recent advancements in drug delivery systems have led to the development of liposomal formulations, which are emerging as ideal carriers for anti-inflammatory agents directly to inflammatory target cells [110]. This enhances local efficacy while minimizing systemic exposure and associated side effects [120]. Among advanced liposomal formulations, invasomes are regarded as promising lipid-based nanocarriers for topical drug delivery, especially in the treatment of psoriasis [121,122]. For instance, Verma et al. investigated the use of cyclosporin-A invasomes for managing cyclosporin A-induced autoimmunity (CsA-AI), a T-cell-mediated inflammatory autoimmune disease that resembles human scleroderma [123]. Ex vivo studies demonstrated significantly higher cyclosporin deposition in deeper skin layers when delivered using invasomes compared to conventional liposomes. It has been demonstrated that increasing terpenes and ethanol in invasomes significantly improved cyclosporine deposition in these deeper layers [124].

Additionally, cyclosporin-A vesicles, formulated using ethanol as one of the components in volume ratios of 10% and 20%, also exhibited enhanced deposition in the stratum corneum when compared with those devoid of ethanol. These findings mark significant progress in the topical delivery of challenging molecules [125].

In another study, Brotzu et al. demonstrated the efficacy of liposomes as a topical application for treating alopecia areata [126]. Their patented lotion aimed at enhancing scalp microcirculation through the vehiculation of di-homo-γ-linolenic acid (DGLA), a precursor of prostaglandin E1 (PGE1), which improves circulation [127]. The formulation also mitigated dihydrotestosterone (DHT) effects through the action of S-Equol, a natural flavonoid that suppresses 5α-reductase activity while promoting metabolism via a carnitine derivative. The active ingredients were encapsulated in phospholipid liposomes to ensure effective delivery [128].

#### 2.2.2. Skin Cancer

Skin cancer is the most prevalent form of malignant neoplasm in humans, and recent statistics indicate a concerning rise in mortality rates associated with this disease [129].

Melanoma, in particular, is recognized as the most deadly type of skin cancer due to the abnormal growth of melanocytes in the epidermis [130].

Over the past few decades, some progress has been made in the treatment of melanoma, with surgical resection in the early stages [131], followed by chemotherapy, radiotherapy, and immunotherapy during metastatic phases [132]. However, these conventional methods often face significant challenges, such as significant side effects, toxicity, and the emergence of drug resistance [64]. Chemotherapeutic agents used to treat melanoma are often limited by systemic toxicity and poor tumor selectivity [133]. Liposomal formulations address these issues by improving drug solubility, enhancing pharmacokinetics, and reducing off-target effects [134]. For instance, Batist et al. demonstrated the topical efficacy of liposomal doxorubicin (Doxil^®^—Johnson & Johnson; New Brunswick, New Jersey, U.S.) in the treatment of melanoma and how encapsulating doxorubicin in liposomes reduced cardiotoxicity while maintaining its antitumor efficacy compared to free doxorubicin [135]. In addition, encapsulated doxorubicin liposomes combined with cyclophosphamide showed a significantly improved antitumor effect in an experimental pulmonary metastatic melanoma mouse model [136].

Another important revolutionary approach in melanoma is represented by immunotherapy, which sees drugs targeting immune checkpoints, such as anti-PD-1 and anti-CTLA-4 antibodies, demonstrating significant promise [137]. However, these therapies can lead to severe immune-related side effects. Researchers have explored liposomes as carriers to enhance the specificity and reduce the toxicity of immunotherapeutic agents. Alimohammadi et al. evaluated the therapeutic efficiency of liposomal anti-CTLA-4 in large established tumors inoculated subcutaneously in a B16 mouse melanoma model. The results indicated that liposomal anti-CTLA-4 not only reduced tumor size but also improved survival rates compared to non-liposomal anti-CTLA-4 [138]. Current therapeutic approaches for melanoma also include photodynamic therapy (PDT), which utilizes photosensitizers activated by light to generate reactive oxygen species (ROS) that kill tumor cells [139]. However, melanoma, in particular melanotic melanoma, exhibits resistance to various treatment modalities, including PDT [140]. Liposomes can enhance the delivery of photosensitizers, improving their stability and tumor selectivity [141]. This was substantiated by Rensen et al., who provided evidence that liposomal formulations of zinc phthalocyanine, a photosensitizer, incubated in pooled plasma resulted in increased tumor accumulation and enhanced photodynamic therapy (PDT) efficacy compared to free photosensitizers [142].

Another innovative approach is represented by gene therapy, which offers several advantages over traditional strategies due to its high specificity and low toxicity [143]. Mutant BRAF genes are commonly expressed in a majority of melanomas, indicating their significant role in melanocyte biology and disease pathology [144]. Gene therapy using small RNA interference (siRNA) targeting BRAF presents a promising avenue for melanoma treatment [145]. Despite this, the efficiency of gene delivery remains a significant challenge, necessitating the use of specific carriers to ensure the successful delivery of exogenous genes or gene-modifying agents into target cells [146]. Furthermore, cancer-targeting liposomes can be engineered by modifying them with ligands that specifically bind to receptors overexpressed in cancer [147]. For instance, Li. L et al. described the development of nucleolin-targeted liposomes guided by the aptamer AS1411, which selectively binds to this overexpressed protein in melanoma cells. This delivery system improves the targeting and internalization of siRNA into melanoma cells while improving the gene silencing efficiency for the mutant BRAF gene associated with this type of cancer [148]. Additionally, liposomes offer reduced toxicity and increased stability compared to traditional treatments, presenting a promising approach for effective and safe melanoma therapy [149].

#### 2.2.3. Wound Care

Wound healing is a complex physiological process that aims to restore the integrity of damaged tissue through a series of well-coordinated phases. These phases include homeostasis, inflammation, proliferation, and remodeling; each one of these plays a crucial role in ensuring effective recovery from injury [150].

During this process, the release of several growth factors is fundamental to promote angiogenesis, stimulate the formation of granulation tissue, and re-epithelization [151].

Among these growth factors, the human epidermal growth factor (hEGF) is particularly significant due to its involvement in multiple stages of wound healing [152]. However, the application of hEGF in dermal therapy for chronic wounds faces several limitations, including proteolytic activity in chronic wounds, its *short* in vivo half-life, challenges related to effective transdermal delivery, and variability in individual patient responses [153]. Ternullo et al. prepared hEGF-containing neutral (NDLs), cationic (CDLs), and anionic (ADLs) deformable liposomes for topical application to explore the impact of the liposomal charge on the mitogenic effect of hEGF. These nanocarriers were characterized by their in vitro and ex vivo release, together with their penetration capabilities. All deformable liposomes exhibited a high hEGF content, with a slightly higher loading capacity observed in anionic deformable liposomes compared to other formulations [154].

Another innovative approach was proposed by Li et al., who developed liposomes encapsulating madecassoside (MA) to promote cutaneous wound healing [155]. Madecassoside is a bioactive compound derived from Centella asiatica, which not only accelerates wound healing but also exhibits antioxidant, antimicrobial, neuroprotective, and anti-ulcer properties [156]. Using a double-emulsion liposomal system, the researchers demonstrated that madecassoside liposomal formulation *significantly enhanced* dermal wound repair in Sprague Dawley rats by improving cell growth and transdermal delivery efficiency [154]. In addition, Günal et al. investigated the effects of liposomal resveratrol (3,5,40-trihydroxy-trans-stilbene) on incisional and excisional wounds. Their study revealed that both 1% and 5% resveratrol liposomal formulations significantly improved granulation tissue formation, epidermal regeneration, and angiogenesis compared to control groups. Histological evaluations confirmed the substantial effectiveness of these formulations in enhancing the healing process [157]. These findings suggest that such liposomal systems serve as a promising solution for improved chronic wound therapy, although their clinical potential requires further investigation.

#### 2.2.4. Skin Infections

The treatment of bacterial infections is increasingly challenged by the global rise of antibiotic-resistant bacteria [158]. This situation necessitates the investigation and development of innovative solutions to effectively manage these infections. Among the promising alternatives, liposomal formulations have emerged as a significant advancement in the treatment of infections, in particular those associated with dermatological conditions [159]. Liposomal formulations enhance drug delivery by encapsulating antimicrobial agents within lipid bilayers. The ability of liposomes to bypass bacterial resistance mechanisms positions them as valuable tools in combating multidrug-resistant pathogens [160].

One notable application of liposomal formulations is in the treatment of skin infections caused by common pathogens such as *Staphylococcus aureus* [161] and *Pseudomonas aeruginosa* [162]. For instance, research conducted by Hajiahmadi et al. investigated the efficacy of liposomal encapsulation of vancomycin [163], an antibiotic commonly used to treat Methicillin-resistant *Staphylococcus aureus* (MRSA) infections [164].

The study demonstrated that vancomycin-loaded liposomes significantly improved antibacterial activity against these pathogens both in vitro and in vivo. Furthermore, these liposomes exhibited no cytotoxicity effects on human epidermoid cell lines and facilitated sustained release at the infection site, leading to superior therapeutic outcomes when compared to free vancomycin [163]. Liposomal formulations are also being explored as delivery systems for antifungal agents [165]. The emergence of *Candida auris*, a multidrug-resistant yeast, poses a serious challenge to public health due to its ability to colonize skin and cause systemic infections [166]. There is an urgent need for new antifungal therapies to manage both superficial and systemic infections caused by this pathogen.

Joromin et. al. developed a novel antifungal agent, PQA-Az-13, which incorporates indazole, pyrrolidine, and arylpiperazine scaffolds with a trifluoromethyl moiety. Given its high hydrophobicity, PQA-Az-13 was encapsulated in cationic liposomes, demonstrating enhanced antifungal activity levels against *Candida auris* both in vitro biofilms and ex vivo skin colonization models [167].

## 3. Gel-Based Formulations

Gel-based formulations are versatile systems that consist of a three-dimensional network capable of entrapping liquid, resulting in a semi-solid consistency. Topical gels are widely used in dermatology for various therapeutic and cosmetic purposes. Their unique formulation allows for the effective delivery of active ingredients while providing specific benefits for different skin conditions.

### 3.1. Structural and Functional Properties of Gel-Based Formulations

**Figure 2:**
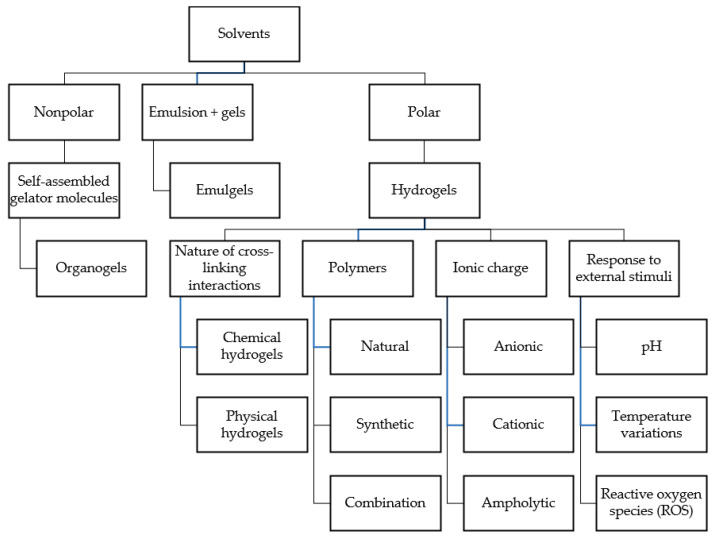
Diagram showing the organization of gels based on their general classification.

Gels are semi-solid formulations formed by the aggregation of colloidal particles in the sol phase, meaning that the particles are bound together to form a network, which confers rigidity to the overall structure [168]. Gels can be categorized into two primary classes based on the chemical composition of their sol phase: organogels and hydrogels [169] (Table 4; Figure 2).

### 3.2. Organogels

Organogels consist of a nonpolar solvent that is immobilized within a three-dimensional network formed by self-assembled gelator molecules (Table 4). This network structure is crucial for the gel-like properties of organogels, as it prevents the flow of the solvent, creating a semi-solid system [170]. The formation of organogels generally occurs through the self-assembly of gelator molecules, driven by non-covalent interactions such as hydrogen bonding and hydrophobic interactions, including van der Waals forces [171]. For example, stigmasterol is considered a low-molecular-weight oleogelator, which forms organogels through intermolecular hydrogen bonding, leading to the creation of a self-assembled network structure when dissolved into appropriate nonpolar solvents such oils, DMSO, or ethanol [172]. Similarly, lecithin organogels are formed when lecithin self-assembles into reverse spherical micelles in nonpolar media, which then aggregate to form a three-dimensional network [173].

### 3.3. Hydrogels

Among gel formulations, hydrogels are the most commonly used and are suitable for a wide range of applications, including drug delivery, wound healing, environmental remediation, and tissue engineering [174,175]. Hydrogels are water-insoluble polymer networks capable of absorbing significant amounts of water or polar solvent due to the presence of hydrophilic groups such as hydroxyl, carboxyl, amine, and sulfate in their structure [176]. They can be obtained starting from natural polymers, such as collagen [177], alginate [178], chitosan [179], gelatin [180], and silk fibroin [181]. Alginate and chitosan are frequently used for drug delivery in cancer treatment [182], while collagen-based hydrogels are widely used in tissue engineering and wound healing [183]. Hyaluronic acid hydrogels serve as excellent moisturizers and, due to their biocompatibility and degradability, are useful for various applications (Figure 2) [184,185].

On the other hand, these systems can also be obtained when synthetic polymers are dispersed in an aqueous phase. Polymers used in hydrogel compositions include polycaprolactone [186], polyvinylpyrrolidone (PVP) [187], polylactic acid (PLA) [188], polyethylene glycol (PEG) [189], and polyvinyl alcohol (PVA) [190], or a combination of these molecules with other natural compounds, as seen when PEG is conjugated with fibrinogen, gelatine, or albumin (Figure 2) [191].

#### 3.3.1. Classification of Hydrogels Based on Cross-Linking and Ionic Charge

Hydrogels can also be categorized into chemical and physical hydrogels based on the nature of the cross-linking interactions that form the three-dimensional polymer network. Chemical hydrogels have permanent junctions composed of covalent cross-linking and polymerizing end-functionalized macromers. Physical hydrogels have transient junctions comprising physical interactions, such as ionic interactions, hydrogen bonding, and crystallization [192].

Hydrogels can also be divided into further groups based on their ionic charge: anionic, cationic, and ampholytic. Due to the presence of charges, these particular hydrogels are sensitive to pH variations. This feature can be used for drug release or to induce a change in the stiffness of the gel network. Some examples of pH-sensitive gels are polyacrylic acid-based gels, which are normally negatively charged [193], while the positive ones can be poly (2-vinyl pyridine) and poly (4-vinyl pyridine), which possess a positive charge due to the presence of nitrogen in the aromatic ring attached to the main chain [194]. Ampholytic hydrogels, which can present both positive and negative electric charges, are created by the copolymerization of anionic and cationic monomers or the incorporation of zwitterionic monomers (e.g., N,N-dimethyl-N-methacryloxyethyl-N-(3-sulphopropyl)-ammonium betaine (SPE)) into the polymer network [195]. These polymers are useful for their ability to exhibit pH-responsive behavior [196], such as increased swelling or contraction, enabling more accurate and controlled drug release (Figure 2) [197].

#### 3.3.2. Responsive Hydrogels

Moreover, it is possible to produce different types of hydrogels that respond to other external stimuli in addition to pH, such as changes in temperature [198]. Thermosensitive hydrogels are especially important in biomedical applications, particularly drug delivery systems. For example, when injected, they can ensure the prolonged release of a specific drug in a localized area. This is achieved through a phase transition mechanism, where the hydrogel’s structure and overall rigidity change upon reaching certain fixed temperatures [199]. These hydrogels are usually composed of polymers such as poly N-isopropylacrylamide, which is sensitive to temperature variations. Another good example is poloxamers, a nonionic type of copolymer characterized by a complex structure composed of a central hydrophobic block made of polypropylene oxide, flanked by two hydrophilic blocks of polyethylene oxide [200].

#### 3.3.3. Therapeutic Role of Hydrogels and Their Active Functionality

Gel formulations may be used not only as carriers but, in some cases, they can cover an active role, directly intervening in the therapeutic activity. Indeed, hydrogels can also contain bonds known to be sensitive to reactive oxygen species (ROS), such as disulfide bonds or di-selenium bonds [201], which can be useful in conditions like inflammation, tumor microenvironments, and neurodegenerative disorders, where ROS levels are elevated [202].

### 3.4. Emulgels

One last derivation that can be considered part of the gel’s family is emulgels. These are hybrid systems that combine the properties of emulsions (water/oil or oil/water) and gels [203]. This dual-phase system allows for the encapsulation of active ingredients within the emulsion while providing the structural integrity and stability of a gel (Figure 2) [204]. Common gelling agents include natural polysaccharides, synthetic polymers, and semi-synthetic agents, which help achieve the desired viscosity and consistency [205].

### 3.5. Therapeutic Applications of Gel-Based Formulations in Dermatology

Gels are highly versatile and adaptable for various dermatological uses, including wound care, infection treatment, post-surgical recovery, reduction of symptoms related to chronic and acute skin inflammation, and the management of skin cancer [206,207]. Gel-based formulations are increasingly recognized as versatile therapeutic agents, spanning topical treatments, advanced drug delivery systems, and controlled-release mechanisms.

Topical gels, and more broadly gels, have long been favored for their ease of application and high patient compliance [208]. Indeed, their non-greasy texture, quick absorption, enhanced spreadability, and cooling effect represent the most known advantages that make this kind of formulation more suitable for reaching both higher patient compliance and therapeutic success [209] (Table 5).

**Table 4:** General classification of gels based on their chemical properties and applications, along with an example of the primary gelling agent used to form each type of gel.

Type	Description	Gelling Agent Examples
*Organogels*	Gels formed with a nonpolar solvent immobilized within a three-dimensional network of gelator molecules	Stigmasterol [172] and lecithin [173]
*“Natural“ Hydrogels*	Water-insoluble natural polymer capable of absorbing significant amounts of water; highly biodegradable and biocompatible	Collagen [177], alginate [178], chitosan [179], gelatin [180], and silk fibroin [181]
*Synthetic Hydrogels*	Gel systems obtained by dispersing synthetic polymers in a liquid phase; more tunable than natural ones	Polycaprolactone [186], PVP [187], PLA [188], PEG [189], and PVA [190]
*Charge-Holder Gels*	Hydrogels categorized based on their ionic charge: anionic, cationic, and ampholytic; commonly used in drug delivery	Polyacrylic acid (anionic) [193], poly (2-vinyl pyridine) (cationic) [194], and SPE (ampholytic) [195]
*Thermosensitive Gels*	Complex systems that respond to temperature changes, often used in drug delivery systems	Poloxamers and poly N-isopropylacrylamide [200]
*ROS-sensitive*	Hydrogels containing bonds sensitive to reactive oxygen species for therapeutic applications	Disulfide bonds and di-selenium bonds [201]
*Hybrid Systems*	Systems combining properties of emulsions and gels for encapsulation of active ingredients while maintaining stability	Natural polysaccharides and synthetic polymers [205]
*Organogels*	Gels formed with a nonpolar solvent immobilized within a three-dimensional network of gelator molecules	Stigmasterol [172] and lecithin [173]

**Table 5:** Summary of the main known advantages of both liposomal and gel formulations on several dermatological conditions.

Dermatological Disease	Advantages of Liposomes in the Treatment of Pathological Conditions	Advantages of Gels in the Treatment of Pathological Conditions
**Inflammatory** **Skin Conditions** *Psoriasis, Atopic* *Dermatitis, and Eczema*	Improved drug penetration and retention in the epidermis and dermis, boosting anti-inflammatory effects while minimizing side effects.	Advanced and enhanced skin penetration; the presence of moisturizing agent helps the overall anti-inflammatory activity of the active ingredients dispersed in the gel formulation.
**Acne Treatment** *Acne Vulgaris*	Liposomal formulations effectively deliver active ingredients to pilosebaceous units, enhancing treatment outcomes compared to traditional methods.	Possibility to incorporate multiple active ingredients (e.g., benzoyl peroxide and clindamycin), enhancing treatment effectiveness. Most of the formulations are oil-free, avoiding the risk of further clogging pores.
**Skin Cancer** **Management** *Basal Cell Carcinoma and Squamous Cell Carcinoma*	Chemotherapeutic agents, such as doxorubicin, are encapsulated to target tumors while minimizing systemic toxicity.	When inserted in a gel formulation, the antineoplastic agents can potentially be released in a more controlled manner, achieving reduced systemic exposure and toxicity.
**Wound Healing** *Chronic Wounds and Burns*	Vesicular systems deliver growth factors (e.g., EGF and TGF-β) and antimicrobial agents to promote tissue repair and reduce infection risks.	Gels can form a physical barrier, reducing wound infections while helping to maintain optimal moisture levels around the wound. These two aspects are critical for a faster healing process.
**Hyperpigmentation** **Disorders** *Melasma and* *Post-Inflammatory* *Hyperpigmentation*	Depigmenting agents (e.g., hydroquinone and kojic acid) in vesicular formulations offer improved stability and targeted action while reducing irritation.	Gel formulations are a suitable and alternative vehicle for the active ingredients used to treat this skin condition, allowing even application and absorption.
**Aesthetic Dermatology** *Anti-Aging Treatments*	Liposomes deliver antioxidants (e.g., vitamin C) and other cosmeceuticals that combat oxidative stress and enhance skin texture.	Injectable gels are being developed for deep injection techniques to lift and rejuvenate aging skin; this formulation is one of the most used in aesthetic medicine.

#### 3.5.1. Skin Inflammation

Even though chronic and severe skin inflammation should be approached with specific therapies related to the pathology to be treated, the use of topical therapies to deal with the most generalized symptoms is considered fundamental to improving patient quality of life [24]. Skin inflammation can last for long periods of time, such as weeks for acute inflammation, and even lasts years in cases of psoriasis, acne, or atopic dermatitis [210].

Hydrogels exhibit significant adaptability to the diverse requirements of pathological skin. This versatility stems from their ability to control drug release, thereby minimizing side effects and maximizing therapeutic efficacy [211]. This enhanced efficacy is achieved through improved penetration of the dispersed or dissolved active ingredient within the viscous hydrogel matrix, potentially leading to a reduction in the overall drug dosage. This concept is exemplified by the work of Gabriel, Doris et al., who demonstrated the superior efficacy of a formulation comprising tacrolimus-loaded nanocarriers dispersed within a Carbopol^®^ (The Lubrizol Corporation; Wickliffe, Ohio USA) [212] hydrogel for the treatment of psoriasis compared to a commercially available paraffin-based product. Furthermore, significantly enhanced skin delivery was observed in an induced psoriatic mouse model utilizing this novel hydrogel formulation [213]. In the past few years, several gel formulations have been developed for the treatment of different inflammatory skin diseases. Inflammatory acne was effectively treated with a combination of adapalene 0.3%/benzoyl peroxide 2.5% loaded into a topical gel in a randomized, double-blind, parallel-group, controlled study [214]. Moreover, an induced alopecia mouse model was effectively treated with an innovative Cyclosporine A and Tempol gel formulation, showing the regeneration of hair follicles and thickened dermis in treated subjects and reduced TNF-α and IL-6 levels, confirming the ability of this gel formulation to deliver an effective topical treatment, without causing typical side effects [215].

#### 3.5.2. Skin Cancer

Skin cancer is regarded as the most prevalent type of carcinoma, with high incidence rates observed globally [216]. It is also considered one of the most dangerous forms of cancer, and for this reason, prevention and early stages diagnosis and treatment are strongly recommended by the scientific community working in this field [217]. Even though in the most serious cases, surgical intervention and/or broad anticancer therapy are the only choices, during the early stages of this pathology, topical treatments can play an essential role [218,219]. The standard treatment typically involves the topical application of imiquimod [220], 5-fluorouracil, and ingenol mebutate. However, prolonged use of these agents can cause side effects ranging from mild to severe, reducing patient compliance and diminishing the likelihood of a successful therapeutic outcome [221,222,223]. Consequently, in recent years, researchers have focused on developing novel formulations featuring innovative carriers and/or antitumoral agents. In this regard, hydrogels and gels have played a pivotal role in advancing therapeutic innovation. Recently, new efficient delivery systems have been developed to ensure the transdermal administration of 5-fluorouracil. Although originally designed for breast cancer treatment, this technology could also be adapted for use in skin cancer therapy. For example, a hydrogel composed of penetration enhancers, like oleic acid and choline glycinate, resulted in effectively delivering the chemotherapeutic drug in efficient anticancer concentrations [224]. Nawaz, Asif et al. designed a 5-fluorouracil nanoparticle formulation dispersed in a chitosan-gelatin-based thermosensitive hydrogel and demonstrated its ability to reduce the dermatological side effects of the drug while increasing its delivery and retention in deeper layers of the skin [225]. Moreover, formulation-compatibility studies of novel skin cancer prevention/treatment molecules such as the toll-like receptor 4 (TLR4) antagonist resatorvid (TAK-242) [226] demonstrated that among different types of formulation, one of the best for drug retention and permeation effectiveness was a gel-based formulation [227].

Gel-based formulations are widely utilized in cancer treatment as carriers for more complex and technically advanced therapies. Recent examples include a 3D microneedle ectoine gel [228], which effectively treated UV-induced melanoma in a rat model; a lipid-based microemulsion incorporated into a Carbopol gel for delivering methotrexate in vitro to squamous epithelial cells [229]; and nanosized imiquimod micelles embedded in a carboxymethyl cellulose gel matrix. Gels have proven to be highly versatile and effective for dermatological applications, demonstrating their adaptability in wound care, infection control, inflammatory skin conditions, and early-stage skin cancer treatment. Their non-greasy texture, rapid absorption, and improved patient compliance make them particularly suitable for a broad range of skin-related therapies.

#### 3.5.3. Wound Care

Wound treatment is a wide field of application in dermatology since it covers superficial lesions, burn injuries, excoriation, and the most severe ones, which involve the deeper layer of the skin. Thanks to their bioavailability and biocompatibility, the involvement of gels in wound care is increasing. For instance, a photo-cross-linked hydrogel made by modified hyaluronic acid and loaded with PR1P (pro-angiogenic prominin-1-binding peptide) was effective and fast in reducing the wounded area on a burn wound model developed on mouse skin due to the effective release of the peptide inserted in the HA matrix [230]. Moreover, gels are suitable as immunomodulators for wound treatment. This effect can be achieved by the components of the formulation, together with the dissolved/dispersed active substances inside of it. Indeed, high-molecular-weight rheological modifiers, such as hyaluronic acid [231] or chitosan [232], can also act as suppressors of the immune response, which is fundamental for chronic wound treatment [233] (e.g., diabetic ulcer and pressure injuries). Additionally, recent research has shown that chitosan combined with alginate salt and poly (ethylene glycol) diacrylate serves as an effective scaffold for treating severe burns. This gel, which is loaded with vascular endothelial cadherin and fibroblast growth factors, demonstrated significant therapeutic effects in excision models, as well as in superficial and severe, deep-burn injury models on mouse skin [234]. These findings, along with the gel’s ability to promote cell growth and exhibit antibacterial activity, indicate that these gel-based formulations are likely to play a crucial role in wound treatment.

#### 3.5.4. Skin Infections

Among dermatological conditions, infections represent one of the most common skin diseases in Europe [235]. Disruptions to the skin barrier, such as those resulting from wounds or chronic skin diseases, like eczema and psoriasis, can compromise its integrity, increasing susceptibility to colonization by a diverse range of microorganisms, including viruses, bacteria, and fungi [236]. Gels in all their declination have been widely used to treat infections due to their ability to retain and deliver antibacterial agents. A notable illustration comes from the research conducted by Abdelghany, Sharif, and colleagues. Their study focused on microneedles infused with ciprofloxacin and embedded in a gel formulation. This innovative approach exhibited remarkable effectiveness in combating Staphylococcus aureus infections. The microneedle system facilitated the delivery of increased antibiotic concentrations to the skin’s deeper layers, significantly reducing the occurrence of unwanted side effects. Gels are also suitable to intervene in fungal infections that are usually characterized by relapses and resistance because of their low solubility, poor skin permeation, and retention [237]. To solve this, antifungal drugs (like luliconazole) can be encapsulated in lipid nanocarriers or formulated in nanocrystals to increase skin penetration. Hydrogel is the most effective vehicle for delivering these formulations due to its high compatibility with both the drug and infected skin [238]. It also exhibits a faster dissolution rate and enhanced antifungal activity compared to more traditional formulations. Moreover, a remarkable subset of peptides has been identified and studied in the past few years, which possesses dual functionality. These molecules not only serve as effective viscosity-enhancing agents in hydrogel formulations but also maintain potent antimicrobial properties, exhibiting both antibacterial and antifungal activities [239]. This unique combination addresses a significant challenge in hydrogel development by eliminating the need to incorporate separate components with varying solubilities to achieve both structural and antimicrobial properties [240,241].

## 4. Limitations and Challenges of Liposomal and Gel Formulations

The development of effective dermatological treatments often relies on refining formulation strategies to enhance efficacy while minimizing side effects. In dermatology, both liposomal and gel formulations meet medical needs and patient requirements effectively; however, they each come with certain limitations.

### 4.1. Liposomal Formulations

While liposomes offer several known benefits, they also present some challenges. The development process is intricate, requiring precise optimization of parameters such as size, charge, and lipid composition, which can increase production costs and complicate regulatory approval processes [242]. Additionally, the complexity of their composition and manufacturing process introduces significant challenges [243]. For example, liposomes often suffer from a short half-life [244], which can result from chemical instability due to the hydrolysis of ester bonds [245] or the oxidation of unsaturated bonds typically present in the acyl chains of phospholipids [80]. Another factor contributing to instability is changes in particle size. The aggregation and fusion of liposomes can lead to increased size and size distribution, ultimately destabilizing the formulation [246]. To address these issues and enhance reproducibility, various methods, such as microfluidic liposomal production, have been explored. While this technique improves reproducibility, the associated costs remain high [247].

Furthermore, while liposomes generally improve drug delivery, some studies have shown that they may occasionally result in excessive systemic absorption, raising concerns about toxicity. Exposure to temperature fluctuations or light can also compromise liposomal integrity, potentially causing premature drug release or degradation. Although the long-term effects of repeated liposome use on the skin remain under investigation, it is known that cationic liposomes can cause toxicity in macrophages and alter the secretion of important immunomodulators [68].

Lastly, the involvement of regulatory agencies, such as the EMA and FDA, can delay the commercialization of liposomal formulations due to the lack of specific guidelines for these types of pharmaceutical products [248]. Liposomal formulations are considered Non-Biological Complex Drugs (NBCDs) [249], requiring extensive characterization of their lipid composition, particle size, zeta potential, and encapsulation efficiency [250]. Moreover, while the FDA issues product-specific guidance (PSG) for generic liposomes (e.g., doxorubicin liposomes), the EMA relies on a 2013 reflection paper, creating uncertainty for developers. An example of this ambivalent situation can be seen in the Lipodox^®^ case: this formulation was the generic version of Doxoil^®^ and was approved by the FDA but rejected by the EMA due to insufficient bioequivalence evidence for free doxorubicin levels, highlighting divergent regional standards that are causing a lack of guidance among the manufacturers [248].

However, it is possible to intervene in most of the problems cited above. Antioxidant molecules (e.g., butylated hydroxytoluene [251]) may be used to avoid the loss of unsaturated bonds, and if this is not possible, these vesicles may be coated with a stabilization enhancer, such as polyethylene glycol [252]. Physical methods are also available for these particles’ stabilization; indeed, the lyophilization [253] technique is demonstrated to increase storage stability and reduce oxidative degradation. Inserting or increasing cholesterol levels in the membrane composition can also help to stabilize the overall membrane’s integrity while avoiding drug leakage by stabilizing the lipid bilayers to external stimuli like temperature change [253].

### 4.2. Gel Formulation

In contrast to liposomal formulations, gel-based systems offer a more straightforward and effective approach to dermatological treatments (Table 6). Gels are highly versatile and can serve as carriers for a wide range of drugs, facilitating their dispersion and application. The manufacturing process for gels is generally less complex than that of liposomes, resulting in reduced production costs. However, gel-based formulations still have limitations that need to be addressed.

While gels provide good surface coverage, their ability to penetrate deeper skin layers is often less effective compared to liposomal formulations. For example, marketed gel formulations tend to retain most of their active ingredients in the upper epidermis, while liposomal gels deliver higher concentrations to the dermis [254]. Additionally, gels may release active ingredients more slowly than other formulations, which can delay therapeutic effects and make them less suitable for conditions that require rapid intervention [255]. To address this issue, penetration enhancers, such as DMSO and ethanol, can be incorporated, although these are often considered irritants [200].

Furthermore, certain components used in gels, such as preservatives and gelators, may cause skin irritation or allergic reactions, particularly in sensitive individuals [256]. The high water content of gels can also increase the risk of microbial contamination if not adequately preserved [257,258]. Stability issues, such as flocculation or syneresis (liquid separation), can compromise their performance and affect the user experience. Finally, environmental factors like temperature, humidity, and light exposure can negatively impact the stability and effectiveness of gel formulations [259,260,261]. Despite the “simpler” nature of these formulations, these last ones face different regulatory challenges too. Unlike oral solid dosage forms, which primarily require bioequivalence testing based on systemic drug levels, topical hydrogels pose additional challenges related to formulation composition, drug release, and skin penetration. Generic versions must match the reference-listed drug (RLD) in terms of spreadability, viscosity, and drug release rate, as these factors influence how the drug is absorbed through the skin. Even small differences in the choice of polymers (e.g., carbomers, xanthan gum, or hyaluronic acid), gelling agents, or preservatives can significantly impact drug diffusion and therapeutic effectiveness [262]. Bioequivalence is another challenge to be faced since the studies that are suitable for these formulations do not rely on traditional pharmacokinetic studies, but instead, alternative methods like in vitro release testing (IVRT) and in vitro permeation testing (IVPT) using human skin models are required to compare the generic formulation to the original. These tests must confirm that the drug release rate and skin penetration profile are equivalent, which can be technically difficult due to variations in skin absorption [263,264].

## 5. Innovations and Emerging Trends

Recent advancements in liposomal formulation technologies have significantly enhanced their therapeutic efficacy, physicochemical stability, and targeted delivery capabilities. Several FDA-approved liposomal and gel-based dermatological products have demonstrated clinical success, providing valuable insights into their efficacy.

Table 7 presents a comparative overview of key FDA-approved liposomal and gel-based products, highlighting their product type, indications, approval status, available clinical data, and therapeutic advantages.

While these FDA-approved products illustrate the clinical benefits of advanced formulations, ongoing research should focus on optimizing patient-specific treatments, enhancing bioavailability, and expanding indications for liposomal and gel-based delivery systems.

Innovations in liposomal preparation methods, particularly microfluidics, have revolutionized the field by enabling precise control over liposome size, polydispersity, and drug encapsulation efficiency [265,266].

Lyophilization has emerged as a critical technique for improving the long-term stability and shelf life of these formulations. This process involves dissolving lipids and therapeutic agents in a monophasic solution, followed by controlled freeze-drying, which results in the formation of liposomes within the nanoscale range (typically 100 to 300 nm) upon rehydration [267]). Additionally, Dual Asymmetric Centrifugation (DAC) has been developed as an innovative approach to producing liposomes with uniform size distribution and enhanced encapsulation efficiency [268,269]. This method utilizes centrifugal forces to facilitate lipid self-assembly, allowing for high reproducibility and scalability in liposome production [268].

Moreover, advances in targeted delivery strategies have led to the development of new formulations, such as cubosomes. These structures offer a higher drug loading capacity due to their larger internal surface area [270] compared to liposomes while also allowing for more controlled drug release [271] and maintaining the biocompatibility and versatility of liposomes [272]. Targeting capabilities have also progressed significantly, with liposomes being modified using specific ligands or antibodies to enable precise drug delivery to cells or tissues. Continued research in this area is expected to further optimize liposomal formulations for clinical applications, improving treatment outcomes across a wide range of diseases.

**Table 7:** Examples of gel and liposomal based formulations aproved by different regulatory agencies with their main indications, clinical data and main indications.

Product Name	Type (Liposomal or Gel Based)	Indication	FDA Approval Status	Clinical Data Available	Comparative Analysis
Lidocaine ointment USP, 5%	Liposomal	Mucosal anesthesia, minor burns	ANDA approved (2019)	Bioequivalence study to reference drug; clinical use for intubation lubrication and sunburn relief	Liposomal delivery enhances penetration and prolongs anesthetic effect compared to non-liposomal alternatives [273]
Abreva^®^ (Docosanol)	Liposomal cream	Herpes labialis	OTC FDA approved (2000)	Median healing time: 4.1 days vs. 4.8 days placebo (*p* = 0.0076); reduces pain, burning, and itching (*p* = 0.002)	Reduces healing time by 18 hrs vs. placebo; targets viral envelope fusion but less effective than Viroxyn [274]
Doxil^®^ (Doxorubicin)	PEGylated liposomal	Cancer therapy	FDA approved (1995)	Progression-free survival of 8.6 months vs. 4.2 months for conventional doxorubicin; reduces cardiotoxicity by 60% [275]	Superior safety profile with reduced cardiotoxicity compared to conventional formulations
Twyneo^®^ (Tretinoin and Benzoyl Protide)	Microencapsulated gel	Acne vulgaris	FDA approved (2021)	Phase 3 trials: 50% IGA success rate vs. 26% for vehicle; prevents benzoyl peroxide–tretinoin interaction	Combines efficacy with reduced irritation due to microencapsulation [276]
Emrosi (DFD-29)	Gel-based	Rosacea	FDA Approved (2024)	Late-stage trials showed superior efficacy in reducing inflammation and redness compared to doxycycline	Demonstrated better safety and efficacy compared to Oracea with no significant safety issues [277,278]
Dapsone Gel (Aczone)	Gel-based	Acne and photo-damage	FDA approved (2008)	A meta-analysis of randomized controlled trials was conducted to analyze the efficacy and adverse events of dapsone gel treatment compared with excipient and other drug therapies	Provides sustained release, reducing irritation and improving tolerability over traditional formulations [279,280]

**Figure 3:**
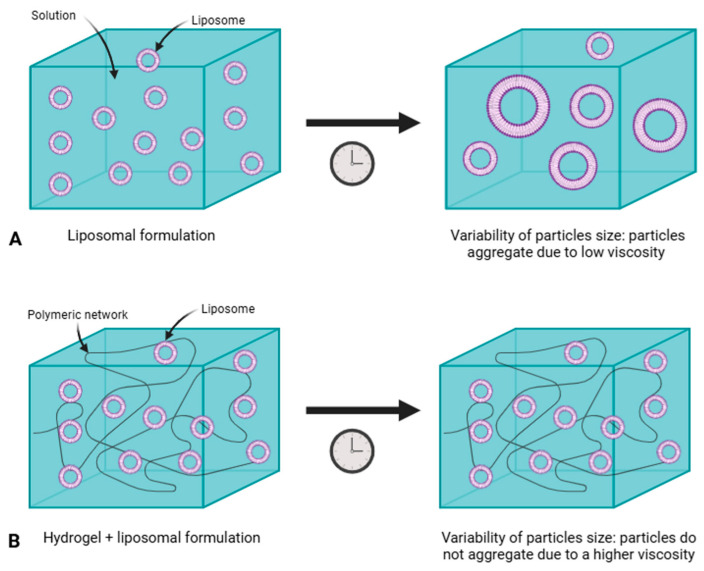
Schematic representation of liposomal stability in presence of hydrogel scaffolds. (**A**) Without hydrogel scaffolds, liposomes tend to aggregate because of environmental factors, like temperature variations, low viscosity, pH changes, or lack of repulsive charges on their surface that may also happen to the solution they are suspended in. (**B**) Liposomes suspended in a hydrogel tend to maintain the same size and do not aggregate due to the polymeric network of the gel, which increases the overall viscosity and delays liposomal aggregation.

Innovations in gel-based formulations are similarly transforming drug delivery and cosmetic technologies. Chemical modifications, such as the addition of carbonyl groups into polysaccharide chains, have expanded the potential applications of these formulations across multiple biomedical fields [281]. This interesting modification can enhance the reactivity of polysaccharides, enabling cross-linking with proteins for tunable hydrogels while also improving biocompatibility, biodegradability, and controlled gelation, making them ideal for biomedical applications like drug delivery and wound healing [281,282]. Furthermore, gels serve as scaffolds for nanoparticle dispersion, including liposomes, to enhance overall stability, efficacy, and penetration [283] (Figure 3). Advancements in technologies like 3D printing have enabled the customization of gel formulations. Semi-solid extrusion techniques now allow for precise control over composition and drug-release kinetics, providing tailored therapeutic solutions [284]. Additionally, environmentally responsive gels [285], such as glucose-sensitive hydrogels, are paving the way for personalized medicine, with promising applications in insulin delivery for diabetes management.

Innovative gel formulations also address urgent medical needs, such as managing hemorrhages. For instance, TRAUMAGEL^®^ [286], a hemostatic gel syringe, contains a sodium alginate and poly(N-acetyl-D-glucosamine, D-glucosamine) hydrogel that expands upon injection, stabilizing hemorrhages temporarily [287]. Many modern gels [288] are designed to be biodegradable and biocompatible, ensuring safety and efficacy across diverse therapeutic contexts [288].

## 6. Conclusions

Liposomal and gel-based formulations represent two of the most innovative and effective drug delivery systems in dermatology, offering significant advantages over conventional topical treatments. Their ability to enhance drug stability, control release profiles, and improve skin penetration makes them valuable tools for treating a wide range of dermatological conditions. Liposomal formulations stand out due to their biocompatibility and structural similarity to biological membranes, allowing them to encapsulate both hydrophilic and lipophilic drugs while facilitating deeper skin penetration. Their ability to reduce systemic absorption when not needed minimizes side effects, making them particularly useful for the localized treatment of inflammatory skin disorders, bacterial and fungal infections, and skin cancers. Additionally, surface modifications, such as ligand attachment or PEGylation, further enhance their targeting capabilities and bioavailability, opening new avenues for personalized medicine and advanced dermatological therapies. Despite these advantages, challenges such as stability issues, formulation complexity, and large-scale production remain key obstacles that require further optimization.

On the other hand, gel-based formulations provide an effective medium for sustained drug release, hydration, and enhanced patient compliance. Their moisturizing and bio-adhesive properties make them particularly beneficial for wound healing, burns, and chronic skin conditions such as eczema and psoriasis. The versatility of gels allows for the incorporation of various bioactive compounds, nanoparticles, and responsive elements, such as thermosensitive or pH-sensitive components, enabling controlled drug release in response to environmental triggers. However, potential drawbacks, including microbial contamination risks, limited penetration depth, and variations in mechanical properties, highlight the need for continued development and refinement.

Recent advancements, including 3D printing, lyophilization techniques, and hybrid formulations combining liposomes with gels, have demonstrated significant potential in enhancing drug stability, prolonging therapeutic effects, and improving precision in drug delivery. By leveraging the unique strengths of both systems, researchers are working toward formulations that optimize treatment efficacy while minimizing side effects and maximizing patient adherence.

It has been demonstrated that both formulations are very interesting and may be among the ones that will hold a fundamental place in patients’ treatment in the future. However, their complexity represents, at the same time, their strength and their weak points. Indeed, stability issues, production cost, and difficulty in scaling up to address market needs, together with a complex regulatory framework, represent huge obstacles for the marked entrance of liposomes and gels. For this reason, they had, and still have, greater success in the cosmetic market, where the regulations are more blurred and clinical trials are not required for market access; nevertheless, in the future, we predict they will play an important role in precision medicine.

Future research should focus on improving the physicochemical stability of liposomes, refining gel formulations for better penetration, and exploring hybrid delivery approaches that integrate both systems for superior therapeutic performance. As the field advances, these formulations are expected to play a central role in the development of next-generation dermatological treatments, addressing the current limitations and expanding clinical applications for a variety of skin conditions.

## Figures and Tables

**Table 2:** Classification of liposomes based on numbers of lipid bilayers composing the micelles (SUVs, LUVs, MLVs, and MVVs).

Type	Description	Preparations Methods
*Small Unilamellar Vesicles (SUVs)*	Composed of a single lipid bilayer surrounding an aqueous compartment, typically sized 20–100 nm	Sonication [72], extrusion [73], and detergent dialysis method [74]
*Large Unilamellar Vesicles (LUVs)*	Enclosed by a single lipid bilayer with a size range of 100–250 nm	Extrusion [75] and dehydration–rehydration [76]
*Multilamellar Vesicles (MLVs)*	Composed of multiple concentric phospholipid bilayers, ranging from 1 to 5 µm in size	Thin-film hydration [77] and reverse-phase evaporation [78]
*Multivesicular Vesicles (MVVs)*	Contain multiple unilamellar vesicles within a larger liposome structure	Cochleate cylinder, interdigitated bilayer, bulk w/wo double emulsion, and microfluidic w/wo emulsion [79]

**Table 6:** Comparison of liposomal and hydrogel drug release mechanisms, along with their main advantages and disadvantages.

Feature	Liposomal Formulations	Gel-Based Formulations
**Mechanism**	Bilayer fusion, destabilization, and enzymatic action	Swelling, diffusion, and matrix erosion
**Release Profile**	Initial burst followed by sustained release	Gradual and prolonged release
**Penetration**	Deep penetration via cell membrane interaction	Localized retention in upper skin layers
**Targeting Ability**	Can be engineered for site-specific delivery	Limited targeting, with mainly localized effect
**Stability**	Susceptible to environmental factors	More stable but prone to microbial contamination
**Applications**	Systemic and localized delivery	Mostly topical, wound healing
**Advantages**	Enhanced skin penetration; enhanced drug delivery; reduced side effects; versatility in formulations; controlled release; and improved stability	Enhanced drug delivery; non-greasy texture; cooling and soothing effect; stability/long shelf life; moisturizing properties; bio adhesive properties; simple composition; and versatility
**Disadvantages**	Complexity in formulations; variability in drug penetration due to environmental factors; physical instability/short shelf life; limited knowledge of long-term effects; and production cost	Partial release of active ingredients; potential irritants (preservatives and few gelling agents); microbial contamination risk; physical stability issues; limited applicability; and limited penetration

## Data Availability

No new data were created or analyzed in this study. Data sharing is not applicable to this article.

## References

[1] Tree C. Skin and Its Appendages. https://clinicalpub.com/skin-and-its-appendages/.

[2] Stander S., Schmelz M. (2024). Skin Innervation. J. Investig. Dermatol..

[3] Nagashima K., Tokizawa K., Marui S. (2018). Thermal comfort. Handb. Clin. Neurol..

[4] Yamasaki K., Gallo R.L. (2008). Antimicrobial peptides in human skin disease. Eur. J. Dermatol..

[5] Ito S., Wakamatsu K. (2011). Diversity of human hair pigmentation as studied by chemical analysis of eumelanin and pheomelanin. J. Eur. Acad. Dermatol. Venereol..

[6] Valeyrie-Allanore L., Sassolas B., Roujeau J.C. (2007). Drug-induced skin, nail and hair disorders. Drug Saf..

[7] Griffiths C.E., van de Kerkhof P., Czarnecka-Operacz M. (2017). Psoriasis and Atopic Dermatitis. Dermatol. Ther. Heidelb..

[8] Leiter U., Eigentler T., Garbe C. (2014). Epidemiology of skin cancer. Adv. Exp. Med. Biol..

[9] Tottoli E.M., Dorati R., Genta I., Chiesa E., Pisani S., Conti B. (2020). Skin Wound Healing Process and New Emerging Technologies for Skin Wound Care and Regeneration. Pharmaceutics.

[10] Tirupathi R., Areti S., Salim S.A., Palabindala V., Jonnalagadda N. (2019). Acute bacterial skin and soft tissue infections: New drugs in ID armamentarium. J. Community Hosp. Intern. Med. Perspect..

[11] Karimkhani C., Dellavalle R.P., Coffeng L.E., Flohr C., Hay R.J., Langan S.M., Nsoesie E.O., Ferrari A.J., Erskine H.E., Silverberg J.I. (2017). Global Skin Disease Morbidity and Mortality: An Update From the Global Burden of Disease Study 2013. JAMA Dermatol..

[12] Song A., Lee S.E., Kim J.H. (2022). Immunopathology and Immunotherapy of Inflammatory Skin Diseases. Immune Netw..

[13] Elahi N., Astaneh M.E., Ai J., Rizwan M. (2024). Atopic dermatitis treatment: A comprehensive review of conventional and novel bioengineered approaches. Int. J. Biol. Macromol..

[14] Langan S.M., Irvine A.D., Weidinger S. (2020). Atopic dermatitis. Lancet.

[15] Hengge U.R., Ruzicka T., Schwartz R.A., Cork M.J. (2006). Adverse effects of topical glucocorticosteroids. J. Am. Acad. Dermatol..

[16] Loden M. (2003). Role of topical emollients and moisturizers in the treatment of dry skin barrier disorders. Am. J. Clin. Dermatol..

[17] Holmes A.D., Steinhoff M. (2017). Integrative concepts of rosacea pathophysiology, clinical presentation and new therapeutics. Exp. Dermatol..

[18] Hoover R.M., Erramouspe J. (2018). Role of Topical Oxymetazoline for Management of Erythematotelangiectatic Rosacea. Ann. Pharmacother..

[19] Armstrong A.W., Read C. (2020). Pathophysiology, Clinical Presentation, and Treatment of Psoriasis: A Review. JAMA.

[20] Rendon A., Schakel K. (2019). Psoriasis Pathogenesis and Treatment. Int. J. Mol. Sci..

[21] Lee H.J., Kim M. (2023). Challenges and Future Trends in the Treatment of Psoriasis. Int. J. Mol. Sci..

[22] Uva L., Miguel D., Pinheiro C., Antunes J., Cruz D., Ferreira J., Filipe P. (2012). Mechanisms of action of topical corticosteroids in psoriasis. Int. J. Endocrinol..

[23] Guo J.W., Jee S.H. (2021). Strategies to Develop a Suitable Formulation for Inflammatory Skin Disease Treatment. Int. J. Mol. Sci..

[24] Ujiie H., Rosmarin D., Schon M.P., Stander S., Boch K., Metz M., Maurer M., Thaci D., Schmidt E., Cole C. (2022). Unmet Medical Needs in Chronic, Non-communicable Inflammatory Skin Diseases. Front. Med..

[25] Canchy L., Kerob D., Demessant A., Amici J.M. (2023). Wound healing and microbiome, an unexpected relationship. J. Eur. Acad. Dermatol. Venereol..

[26] Gouin J.P., Kiecolt-Glaser J.K. (2011). The impact of psychological stress on wound healing: Methods and mechanisms. Immunol. Allergy Clin. N. Am..

[27] Guo S., Dipietro L.A. (2010). Factors affecting wound healing. J. Dent. Res..

[28] Takeo M., Lee W., Ito M. (2015). Wound healing and skin regeneration. Cold Spring Harb. Perspect. Med..

[29] Gosain A., DiPietro L.A. (2004). Aging and wound healing. World J. Surg..

[30] Schilrreff P., Alexiev U. (2022). Chronic Inflammation in Non-Healing Skin Wounds and Promising Natural Bioactive Compounds Treatment. Int. J. Mol. Sci..

[31] Ding X., Tang Q., Xu Z., Xu Y., Zhang H., Zheng D., Wang S., Tan Q., Maitz J., Maitz P.K. (2022). Challenges and innovations in treating chronic and acute wound infections: From basic science to clinical practice. Burns Trauma.

[32] Mirhaj M., Labbaf S., Tavakoli M., Seifalian A.M. (2022). Emerging treatment strategies in wound care. Int. Wound J..

[33] Frykberg R.G., Banks J. (2015). Challenges in the Treatment of Chronic Wounds. Adv. Wound Care.

[34] Kim R.H., Armstrong A.W. (2012). Nonmelanoma skin cancer. Dermatol. Clin..

[35] Didona D., Paolino G., Bottoni U., Cantisani C. (2018). Non Melanoma Skin Cancer Pathogenesis Overview. Biomedicines.

[36] Kim Y., He Y.Y. (2014). Ultraviolet radiation-induced non-melanoma skin cancer: Regulation of DNA damage repair and inflammation. Genes Dis..

[37] Saginala K., Barsouk A., Aluru J.S., Rawla P., Barsouk A. (2021). Epidemiology of Melanoma. Med. Sci..

[38] Ostrowski S.M., Fisher D.E. (2021). Biology of Melanoma. Hematol. Oncol. Clin. N. Am..

[39] Anna B., Blazej Z., Jacqueline G., Andrew C.J., Jeffrey R., Andrzej S. (2007). Mechanism of UV-related carcinogenesis and its contribution to nevi/melanoma. Expert Rev. Dermatol..

[40] Davis L.E., Shalin S.C., Tackett A.J. (2019). Current state of melanoma diagnosis and treatment. Cancer Biol. Ther..

[41] Naseri H., Safaei A.A. (2025). Diagnosis and prognosis of melanoma from dermoscopy images using machine learning and deep learning: A systematic literature review. BMC Cancer.

[42] Grice E.A., Segre J.A. (2011). The skin microbiome. Nat. Rev. Microbiol..

[43] Skowron K., Bauza-Kaszewska J., Kraszewska Z., Wiktorczyk-Kapischke N., Grudlewska-Buda K., Kwiecinska-Pirog J., Walecka-Zacharska E., Radtke L., Gospodarek-Komkowska E. (2021). Human Skin Microbiome: Impact of Intrinsic and Extrinsic Factors on Skin Microbiota. Microorganisms.

[44] Aly R., Baron S. (1996). Microbial Infections of Skin and Nails. Medical Microbiology.

[45] Sandoval A.G.W., Vaughn L.T., Huang J.T., Barbieri J.S. (2023). Role of Tumor Necrosis Factor-alpha Inhibitors in the Treatment and Occurrence of Acne: A Systematic Review. JAMA Dermatol..

[46] Ianiri G., LeibundGut-Landmann S., Dawson T.L. (2022). Malassezia: A Commensal, Pathogen, and Mutualist of Human and Animal Skin. Annu. Rev. Microbiol..

[47] Ki V., Rotstein C. (2008). Bacterial skin and soft tissue infections in adults: A review of their epidemiology, pathogenesis, diagnosis, treatment and site of care. Can. J. Infect. Dis. Med. Microbiol..

[48] Zhou S., Yao Z. (2022). Roles of Infection in Psoriasis. Int. J. Mol. Sci..

[49] Spada F., Barnes T.M., Greive K.A. (2019). Emollient formulations containing antiseptics reduce effectively the level of Staphylococcus aureus on skin. Clin. Cosmet. Investig. Dermatol..

[50] Lei V., Petty A.J., Atwater A.R., Wolfe S.A., MacLeod A.S. (2020). Skin Viral Infections: Host Antiviral Innate Immunity and Viral Immune Evasion. Front. Immunol..

[51] Whitley R.J. (2002). Herpes simplex virus infection. Semin. Pediatr. Infect. Dis..

[52] Rana H., Truong N.R., Sirimanne D.R., Cunningham A.L. (2024). Breaching the Barrier: Investigating Initial Herpes Simplex Viral Infection and Spread in Human Skin and Mucosa. Viruses.

[53] Kennedy P.G.E., Gershon A.A. (2018). Clinical Features of Varicella-Zoster Virus Infection. Viruses.

[54] Wolf J., Kist L.F., Pereira S.B., Quessada M.A., Petek H., Pille A., Maccari J.G., Mutlaq M.P., Nasi L.A. (2024). Human papillomavirus infection: Epidemiology, biology, host interactions, cancer development, prevention, and therapeutics. Rev. Med. Virol..

[55] Trizna Z. (2002). Viral diseases of the skin: Diagnosis and antiviral treatment. Paediatr. Drugs.

[56] Nazarko L. (2010). Fungal skin infections and HCAs: Identify, treat and act. Br. J. Healthc. Assist..

[57] Hube B., Hay R., Brasch J., Veraldi S., Schaller M. (2015). Dermatomycoses and inflammation: The adaptive balance between growth, damage, and survival. J. Mycol. Med..

[58] Howell S.A. (2023). Dermatopathology and the Diagnosis of Fungal Infections. Br. J. Biomed. Sci..

[59] Tuor M., LeibundGut-Landmann S. (2023). The skin mycobiome and intermicrobial interactions in the cutaneous niche. Curr. Opin. Microbiol..

[60] Limper A.H., Adenis A., Le T., Harrison T.S. (2017). Fungal infections in HIV/AIDS. Lancet Infect. Dis..

[61] Kumar M., Saadaoui M., Al Khodor S. (2022). Infections and Pregnancy: Effects on Maternal and Child Health. Front. Cell Infect. Microbiol..

[62] Frasca D., Strbo N. (2022). Effects of Obesity on Infections with Emphasis on Skin Infections and Wound Healing. J. Dermatol. Skin Sci..

[63] Kyle A.A., Dahl M.V. (2004). Topical therapy for fungal infections. Am. J. Clin. Dermatol..

[64] Ezike T.C., Okpala U.S., Onoja U.L., Nwike C.P., Ezeako E.C., Okpara O.J., Okoroafor C.C., Eze S.C., Kalu O.L., Odoh E.C. (2023). Advances in drug delivery systems, challenges and future directions. Heliyon.

[65] Neupane R., Boddu S.H.S., Renukuntla J., Babu R.J., Tiwari A.K. (2020). Alternatives to Biological Skin in Permeation Studies: Current Trends and Possibilities. Pharmaceutics.

[66] Lee M.K. (2020). Liposomes for Enhanced Bioavailability of Water-Insoluble Drugs: In Vivo Evidence and Recent Approaches. Pharmaceutics.

[67] Verma P., Pathak K. (2010). Therapeutic and cosmeceutical potential of ethosomes: An overview. J. Adv. Pharm. Technol. Res..

[68] Inglut C.T., Sorrin A.J., Kuruppu T., Vig S., Cicalo J., Ahmad H., Huang H.C. (2020). Immunological and Toxicological Considerations for the Design of Liposomes. Nanomaterials.

[69] Nsairat H., Khater D., Sayed U., Odeh F., Al Bawab A., Alshaer W. (2022). Liposomes: Structure, composition, types, and clinical applications. Heliyon.

[70] Thang N.H., Chien T.B., Cuong D.X. (2023). Polymer-Based Hydrogels Applied in Drug Delivery: An Overview. Gels.

[71] Akbarzadeh A., Rezaei-Sadabady R., Davaran S., Joo S.W., Zarghami N., Hanifehpour Y., Samiei M., Kouhi M., Nejati-Koshki K. (2013). Liposome: Classification, preparation, and applications. Nanoscale Res. Lett..

[72] de Freitas C.F., Calori I.R., Tessaro A.L., Caetano W., Hioka N. (2019). Rapid formation of Small Unilamellar Vesicles (SUV) through low-frequency sonication: An innovative approach. Colloids Surf. B Biointerfaces.

[73] Ong S.G., Chitneni M., Lee K.S., Ming L.C., Yuen K.H. (2016). Evaluation of Extrusion Technique for Nanosizing Liposomes. Pharmaceutics.

[74] Zhong Q., Zhang H. (2023). Preparation of Small Unilamellar Vesicle Liposomes Using Detergent Dialysis Method. Methods Mol. Biol..

[75] Lapinski M.M., Castro-Forero A., Greiner A.J., Ofoli R.Y., Blanchard G.J. (2007). Comparison of liposomes formed by sonication and extrusion: Rotational and translational diffusion of an embedded chromophore. Langmuir.

[76] Seltzer S.E., Gregoriadis G., Dick R. (1988). Evaluation of the dehydration-rehydration method for production of contrast-carrying liposomes. Investig. Radiol..

[77] Xiang B., Cao D.Y. (2018). Preparation of Drug Liposomes by Thin-Film Hydration and Homogenization.

[78] Pidgeon C., McNeely S., Schmidt T., Johnson J.E. (1987). Multilayered vesicles prepared by reverse-phase evaporation: Liposome structure and optimum solute entrapment. Biochemistry.

[79] Giuliano C.B., Cvjetan N., Ayache J., Walde P. (2021). Multivesicular Vesicles: Preparation and Applications. ChemSystemsChem.

[80] Anderson M., Omri A. (2004). The effect of different lipid components on the in vitro stability and release kinetics of liposome formulations. Drug Deliv..

[81] Machin J.M., Kalli A.C., Ranson N.A., Radford S.E. (2023). Protein-lipid charge interactions control the folding of outer membrane proteins into asymmetric membranes. Nat. Chem..

[82] Li Y., Wang J., Gao Y., Zhu J., Wientjes M.G., Au J.L. (2011). Relationships between liposome properties, cell membrane binding, intracellular processing, and intracellular bioavailability. AAPS J..

[83] Rezagholizade-Shirvan A., Soltani M., Shokri S., Radfar R., Arab M., Shamloo E. (2024). Bioactive compound encapsulation: Characteristics, applications in food systems, and implications for human health. Food Chem. X.

[84] Abd El-Alim S.H., Kassem A.A., Basha M., Salama A. (2019). Comparative study of liposomes, ethosomes and transfersomes as carriers for enhancing the transdermal delivery of diflunisal: In vitro and in vivo evaluation. Int. J. Pharm..

[85] Natsheh H., Touitou E. (2020). Phospholipid Vesicles for Dermal/Transdermal and Nasal Administration of Active Molecules: The Effect of Surfactants and Alcohols on the Fluidity of Their Lipid Bilayers and Penetration Enhancement Properties. Molecules.

[86] Opatha S.A.T., Titapiwatanakun V., Chutoprapat R. (2020). Transfersomes: A Promising Nanoencapsulation Technique for Transdermal Drug Delivery. Pharmaceutics.

[87] Abdulbaqi I.M., Darwis Y., Khan N.A., Assi R.A., Khan A.A. (2016). Ethosomal nanocarriers: The impact of constituents and formulation techniques on ethosomal properties, in vivo studies, and clinical trials. Int. J. Nanomed..

[88] Kuksis A. (1992). Yolk lipids. Biochim. Biophys. Acta.

[89] Nkanga C.I., Krause R.W., Noundou X.S., Walker R.B. (2017). Preparation and characterization of isoniazid-loaded crude soybean lecithin liposomes. Int. J. Pharm..

[90] Luo D., Li N., Carter K.A., Lin C., Geng J., Shao S., Huang W.C., Qin Y., Atilla-Gokcumen G.E., Lovell J.F. (2016). Rapid Light-Triggered Drug Release in Liposomes Containing Small Amounts of Unsaturated and Porphyrin-Phospholipids. Small.

[91] van Hoogevest P., Wendel A. (2014). The use of natural and synthetic phospholipids as pharmaceutical excipients. Eur. J. Lipid Sci. Technol..

[92] Marquez M.G., Dotson R., Pias S., Frolova L.V., Tartis M.S. (2020). Phospholipid prodrug conjugates of insoluble chemotherapeutic agents for ultrasound targeted drug delivery. Nanotheranostics.

[93] Purusottam R.N., Senicourt L., Lacapere J.J., Tekely P. (2015). Probing the gel to liquid-crystalline phase transition and relevant conformation changes in liposomes by (13)C magic-angle spinning NMR spectroscopy. Biochim. Biophys. Acta.

[94] Redondo-Morata L., Giannotti M.I., Sanz F. (2012). Influence of cholesterol on the phase transition of lipid bilayers: A temperature-controlled force spectroscopy study. Langmuir.

[95] Neunert G., Tomaszewska-Gras J., Baj A., Gauza-Wlodarczyk M., Witkowski S., Polewski K. (2021). Phase Transitions and Structural Changes in DPPC Liposomes Induced by a 1-Carba-Alpha-Tocopherol Analogue. Molecules.

[96] Abbasi H., Kouchak M., Mirveis Z., Hajipour F., Khodarahmi M., Rahbar N., Handali S. (2023). What We Need to Know about Liposomes as Drug Nanocarriers: An Updated Review. Adv. Pharm. Bull..

[97] Ohradanova-Repic A., Nogueira E., Hartl I., Gomes A.C., Preto A., Steinhuber E., Muhlgrabner V., Repic M., Kuttke M., Zwirzitz A. (2018). Fab antibody fragment-functionalized liposomes for specific targeting of antigen-positive cells. Nanomedicine.

[98] Wiedenhoeft T., Tarantini S., Nyul-Toth A., Yabluchanskiy A., Csipo T., Balasubramanian P., Lipecz A., Kiss T., Csiszar A., Csiszar A. (2019). Fusogenic liposomes effectively deliver resveratrol to the cerebral microcirculation and improve endothelium-dependent neurovascular coupling responses in aged mice. Geroscience.

[99] Sun W.-X., Zhang C.-t., Yu X.-N., Guo J.-b., Ma H., Liu K., Luo P., Ren J. (2022). Preparation and pharmacokinetic study of diosmetin long-circulating liposomes modified with lactoferrin. J. Funct. Foods.

[100] Ju C., Zhang C. (2018). Preparation and Characterization of pH Sensitive Drug Liposomes.

[101] Lee Y., Thompson D.H. (2017). Stimuli-responsive liposomes for drug delivery. Wiley Interdiscip. Rev. Nanomed. Nanobiotechnol..

[102] Haemmerich D., Ramajayam K.K., Newton D.A. (2023). Review of the Delivery Kinetics of Thermosensitive Liposomes. Cancers.

[103] Ho E.A., Ramsay E., Ginj M., Anantha M., Bregman I., Sy J., Woo J., Osooly-Talesh M., Yapp D.T., Bally M.B. (2010). Characterization of cationic liposome formulations designed to exhibit extended plasma residence times and tumor vasculature targeting properties. J. Pharm. Sci..

[104] Ashrafizadeh M., Delfi M., Zarrabi A., Bigham A., Sharifi E., Rabiee N., Paiva-Santos A.C., Kumar A.P., Tan S.C., Hushmandi K. (2022). Stimuli-responsive liposomal nanoformulations in cancer therapy: Pre-clinical & clinical approaches. J. Control. Release.

[105] Petrilli R., Eloy J.O., Lee R.J., Lopez R.F.V. (2018). Preparation of Immunoliposomes by Direct Coupling of Antibodies Based on a Thioether Bond. Methods Mol. Biol..

[106] Wang N., Chen M., Wang T. (2019). Liposomes used as a vaccine adjuvant-delivery system: From basics to clinical immunization. J. Control Release.

[107] Nsairat H., Alshaer W., Odeh F., Esawi E., Khater D., Bawab A.A., El-Tanani M., Awidi A., Mubarak M.S. (2023). Recent advances in using liposomes for delivery of nucleic acid-based therapeutics. OpenNano.

[108] Dejeu I.L., Vicas L.G., Marian E., Ganea M., Frent O.D., Maghiar P.B., Bodea F.I., Dejeu G.E. (2024). Innovative Approaches to Enhancing the Biomedical Properties of Liposomes. Pharmaceutics.

[109] de la Maza A., Coderch L., Lopez O., Baucells J., Parra J.L. (1997). Permeability changes caused by surfactants in liposomes that model the stratum corneum lipid composition. J. Am. Oil Chem. Soc..

[110] van Alem C.M.A., Metselaar J.M., van Kooten C., Rotmans J.I. (2021). Recent Advances in Liposomal-Based Anti-Inflammatory Therapy. Pharmaceutics.

[111] Tran M.A., Watts R.J., Robertson G.P. (2009). Use of liposomes as drug delivery vehicles for treatment of melanoma. Pigment. Cell Melanoma Res..

[112] Khasawneh D.M., Oweis R.J., Alsmadi M. (2024). A Comprehensive Analysis of Liposomal-Based Nanocarriers for Treating Skin and Soft Tissue Infection. Curr. Drug Deliv..

[113] Puccetti M., Pariano M., Schoubben A., Giovagnoli S., Ricci M. (2024). Biologics, theranostics, and personalized medicine in drug delivery systems. Pharmacol. Res..

[114] Sercombe L., Veerati T., Moheimani F., Wu S.Y., Sood A.K., Hua S. (2015). Advances and Challenges of Liposome Assisted Drug Delivery. Front. Pharmacol..

[115] Faye O., Flohr C., Kabashima K., Ma L., Paller A.S., Rapelanoro F.R., Steinhoff M., Su J.C., Takaoka R., Wollenberg A. (2024). Atopic dermatitis: A global health perspective. J. Eur. Acad. Dermatol. Venereol..

[116] Sibbald C. (2023). Alopecia Areata: An Updated Review for 2023. J. Cutan Med. Surg..

[117] Di Agosta E., Salvati L., Corazza M., Baiardini I., Ambrogio F., Angileri L., Antonelli E., Belluzzo F., Bonamonte D., Bonzano L. (2021). Quality of life in patients with allergic and immunologic skin diseases: In the eye of the beholder. Clin. Mol. Allergy.

[118] Ferrucci S.M., Tavecchio S., Marzano A.V., Buffon S. (2023). Emerging Systemic Treatments for Atopic Dermatitis. Dermatol. Ther. Heidelb..

[119] Lima X.T., Seidler E.M., Lima H.C., Kimball A.B. (2009). Long-term safety of biologics in dermatology. Dermatol. Ther..

[120] Laniado-Laborin R., Cabrales-Vargas M.N. (2009). Amphotericin B: Side effects and toxicity. Rev. Iberoam. Micol..

[121] Akombaetwa N., Ilangala A.B., Thom L., Memvanga P.B., Witika B.A., Buya A.B. (2023). Current Advances in Lipid Nanosystems Intended for Topical and Transdermal Drug Delivery Applications. Pharmaceutics.

[122] Samir B., El-Kamel A., Zahran N., Heikal L. (2024). Resveratrol-loaded invasome gel: A promising nanoformulation for treatment of skin cancer. Drug Deliv. Transl. Res..

[123] Verma D.D., Fahr A. (2004). Synergistic penetration enhancement effect of ethanol and phospholipids on the topical delivery of cyclosporin A. J. Control. Release.

[124] Kumar R., Dogra S., Amarji B., Singh B., Kumar S., Sharma, Vinay K., Mahajan R., Katare O.P. (2016). Efficacy of Novel Topical Liposomal Formulation of Cyclosporine in Mild to Moderate Stable Plaque Psoriasis: A Randomized Clinical Trial. JAMA Dermatol..

[125] Batzri S., Korn E.D. (1973). Single bilayer liposomes prepared without sonication. Biochim. Biophys. Acta.

[126] Brotzu G., Fadda A.M., Manca M.L., Manca T., Marongiu F., Campisi M., Consolaro F. (2019). A liposome-based formulation containing equol, dihomo-gamma-linolenic acid and propionyl-l-carnitine to prevent and treat hair loss: A prospective investigation. Dermatol. Ther..

[127] Thum J., Caspary L., Creutzig A., Alexander K. (1998). Intra-arterial and intravenous administration of prostaglandin E1 cause different changes to skin microcirculation in patients with peripheral arterial occlusive disease. Vasa.

[128] Ashtikar M., Nagarsekar K., Fahr A. (2016). Transdermal delivery from liposomal formulations—Evolution of the technology over the last three decades. J. Control Release.

[129] Roky A.H., Islam M.M., Ahasan A.M.F., Mostaq M.S., Mahmud M.Z., Amin M.N., Mahmud M.A. (2024). Overview of skin cancer types and prevalence rates across continents. Cancer Pathog. Ther..

[130] Ali Z., Yousaf N., Larkin J. (2013). Melanoma epidemiology, biology and prognosis. EJC Suppl..

[131] Elder D. (1999). Tumor progression, early diagnosis and prognosis of melanoma. Acta Oncol..

[132] Garbe C., Eigentler T.K. (2007). Diagnosis and treatment of cutaneous melanoma: State of the art 2006. Melanoma Res..

[133] Hsan K.M., Chen C.C., Shyur L.F. (2010). Current research and development of chemotherapeutic agents for melanoma. Cancers.

[134] Matos C.M. (2024). Special Issue “Application Progress of Liposomes in Drug Development”. Int. J. Mol. Sci..

[135] Batist G., Ramakrishnan G., Rao C.S., Chandrasekharan A., Gutheil J., Guthrie T., Shah P., Khojasteh A., Nair M.K., Hoelzer K. (2001). Reduced cardiotoxicity and preserved antitumor efficacy of liposome-encapsulated doxorubicin and cyclophosphamide compared with conventional doxorubicin and cyclophosphamide in a randomized, multicenter trial of metastatic breast cancer. J. Clin. Oncol..

[136] Shiraga E., Barichello J.M., Ishida T., Kiwada H. (2008). A metronomic schedule of cyclophosphamide combined with PEGylated liposomal doxorubicin has a highly antitumor effect in an experimental pulmonary metastatic mouse model. Int. J. Pharm..

[137] Seidel J.A., Otsuka A., Kabashima K. (2018). Anti-PD-1 and Anti-CTLA-4 Therapies in Cancer: Mechanisms of Action, Efficacy, and Limitations. Front. Oncol..

[138] Gu Z., Da Silva C.G., Van der Maaden K., Ossendorp F., Cruz L.J. (2020). Liposome-Based Drug Delivery Systems in Cancer Immunotherapy. Pharmaceutics.

[139] Shi X., Zhang C.Y., Gao J., Wang Z. (2019). Recent advances in photodynamic therapy for cancer and infectious diseases. Wiley Interdiscip Rev. Nanomed. Nanobiotechnol..

[140] Li X.Y., Tan L.C., Dong L.W., Zhang W.Q., Shen X.X., Lu X., Zheng H., Lu Y.G. (2020). Susceptibility and Resistance Mechanisms During Photodynamic Therapy of Melanoma. Front. Oncol..

[141] Ghosh S., Carter K.A., Lovell J.F. (2019). Liposomal formulations of photosensitizers. Biomaterials.

[142] Rensen P.C., Love W.G., Taylor P.W. (1994). In vitro interaction of zinc(II)-phthalocyanine-containing liposomes and plasma lipoproteins. J. Photochem. Photobiol. B.

[143] Goncalves G.A.R., Paiva R.M.A. (2017). Gene therapy: Advances, challenges and perspectives. Einstein.

[144] Pollock P.M., Harper U.L., Hansen K.S., Yudt L.M., Stark M., Robbins C.M., Moses T.Y., Hostetter G., Wagner U., Kakareka J. (2003). High frequency of BRAF mutations in nevi. Nat. Genet..

[145] Zhang J., Chen B., Gan C., Sun H., Zhang J., Feng L. (2023). A Comprehensive Review of Small Interfering RNAs (siRNAs): Mechanism, Therapeutic Targets, and Delivery Strategies for Cancer Therapy. Int. J. Nanomed..

[146] Kaneda Y. (2000). Virosomes: Evolution of the liposome as a targeted drug delivery system. Adv. Drug Deliv. Rev..

[147] Cho K., Wang X., Nie S., Chen Z.G., Shin D.M. (2008). Therapeutic nanoparticles for drug delivery in cancer. Clin. Cancer Res..

[148] Li L., Hou J., Liu X., Guo Y., Wu Y., Zhang L., Yang Z. (2014). Nucleolin-targeting liposomes guided by aptamer AS1411 for the delivery of siRNA for the treatment of malignant melanomas. Biomaterials.

[149] Golestani P. (2024). Lipid-based nanoparticles as a promising treatment for the skin cancer. Heliyon.

[150] Singh S., Young A., McNaught C.-E. (2017). The physiology of wound healing. Surgery.

[151] Mofazzal Jahromi M.A., Sahandi Zangabad P., Moosavi Basri S.M., Sahandi Zangabad K., Ghamarypour A., Aref A.R., Karimi M., Hamblin M.R. (2018). Nanomedicine and advanced technologies for burns: Preventing infection and facilitating wound healing. Adv. Drug Deliv. Rev..

[152] Demidova-Rice T.N., Hamblin M.R., Herman I.M. (2012). Acute and impaired wound healing: Pathophysiology and current methods for drug delivery, part 2: Role of growth factors in normal and pathological wound healing: Therapeutic potential and methods of delivery. Adv. Skin Wound Care.

[153] Boateng J., Catanzano O. (2015). Advanced Therapeutic Dressings for Effective Wound Healing—A Review. J. Pharm. Sci..

[154] Ternullo S., Basnet P., Holsaeter A.M., Flaten G.E., de Weerd L., Skalko-Basnet N. (2018). Deformable liposomes for skin therapy with human epidermal growth factor: The effect of liposomal surface charge. Eur. J. Pharm. Sci..

[155] Li Z., Liu M., Wang H., Du S. (2016). Increased cutaneous wound healing effect of biodegradable liposomes containing madecassoside: Preparation optimization, in vitro dermal permeation, and in vivo bioevaluation. Int. J. Nanomed..

[156] Bian D., Liu M., Li Y., Xia Y., Gong Z., Dai Y. (2012). Madecassoside, a triterpenoid saponin isolated from Centella asiatica herbs, protects endothelial cells against oxidative stress. J. Biochem. Mol. Toxicol..

[157] Günal M.Y., Ayla Ş., Bedri N., Beker M.Ç., Çağlayan A.B., Aslan İ., Özdemir E.M., Yeşilada E., Kılıç Ü. (2019). The effects of topical liposomal resveratrol on incisional and excisional wound healing process. Turkderm.

[158] Chinemerem Nwobodo D., Ugwu M.C., Oliseloke Anie C., Al-Ouqaili M.T.S., Chinedu Ikem J., Victor Chigozie U., Saki M. (2022). Antibiotic resistance: The challenges and some emerging strategies for tackling a global menace. J. Clin. Lab. Anal..

[159] Rizkita L.D., Putri R.G.P., Farid M., Rizkawati M., Wikaningtyas P. (2024). Liposome drug delivery in combating the widespread topical antibiotic resistance: A narrative review. Beni-Suef Univ. J. Basic Appl. Sci..

[160] Panthi V.K., Fairfull-Smith K.E., Islam N. (2024). Liposomal drug delivery strategies to eradicate bacterial biofilms: Challenges, recent advances, and future perspectives. Int. J. Pharm..

[161] Del Giudice P. (2020). Skin Infections Caused by Staphylococcus aureus. Acta Derm. Venereol..

[162] Spernovasilis N., Psichogiou M., Poulakou G. (2021). Skin manifestations of Pseudomonas aeruginosa infections. Curr. Opin. Infect. Dis..

[163] Hajiahmadi F., Alikhani M.Y., Shariatifar H., Arabestani M.R., Ahmadvand D. (2019). The bactericidal effect of liposomal vancomycin as a topical combating system against Methicillin-resistant Staphylococcus aureus skin wound infection in mice. Med. J. Islam. Repub. Iran.

[164] Rajan S. (2012). Skin and soft-tissue infections: Classifying and treating a spectrum. Cleve Clin. J. Med..

[165] Ayatollahi Mousavi S.A., Mokhtari A., Barani M., Izadi A., Amirbeigi A., Ajalli N., Amanizadeh A., Hadizadeh S. (2023). Advances of liposomal mediated nanocarriers for the treatment of dermatophyte infections. Heliyon.

[166] Forsberg K., Woodworth K., Walters M., Berkow E.L., Jackson B., Chiller T., Vallabhaneni S. (2019). Erratum: Candida auris: The recent emergence of a multidrug-resistant fungal Pathogen. Med. Mycol..

[167] Jaromin A., Zarnowski R., Markowski A., Zagorska A., Johnson C.J., Etezadi H., Kihara S., Mota-Santiago P., Nett J.E., Boyd B.J. (2024). Liposomal formulation of a new antifungal hybrid compound provides protection against Candida auris in the ex vivo skin colonization model. Antimicrob. Agents Chemother..

[168] Tofanica B.M., Belosinschi D., Volf I. (2022). Gels, Aerogels and Hydrogels: A Challenge for the Cellulose-Based Product Industries. Gels.

[169] Chelu M., Musuc A.M. (2023). Polymer Gels: Classification and Recent Developments in Biomedical Applications. Gels.

[170] Rushikesh Khemnar S.S., Nikhil S., Pallavi K., Avdhut K., Vitthal D. (2024). Review Article On Organogels Methodology And Types Of Organogelators. Int. J. Pharm. Sci.

[171] Tang C., Wan Z., Chen Y., Tang Y., Fan W., Cao Y., Song M., Qin J., Xiao H., Guo S. (2022). Structure and Properties of Organogels Prepared from Rapeseed Oil with Stigmasterol. Foods.

[172] Bag B.G., Barai A.C. (2020). Self-assembly of naturally occurring stigmasterol in liquids yielding a fibrillar network and gel. RSC Adv..

[173] Raut S., Bhadoriya S.S., Uplanchiwar V., Mishra V., Gahane A., Jain S.K. (2012). Lecithin organogel: A unique micellar system for the delivery of bioactive agents in the treatment of skin aging. Acta Pharm. Sin. B.

[174] Mantha S., Pillai S., Khayambashi P., Upadhyay A., Zhang Y., Tao O., Pham H.M., Tran S.D. (2019). Smart Hydrogels in Tissue Engineering and Regenerative Medicine. Materials.

[175] Rando G., Scalone E., Sfameni S., Plutino M.R. (2024). Functional Bio-Based Polymeric Hydrogels for Wastewater Treatment: From Remediation to Sensing Applications. Gels.

[176] Bashir S., Hina M., Iqbal J., Rajpar A.H., Mujtaba M.A., Alghamdi N.A., Wageh S., Ramesh K., Ramesh S. (2020). Fundamental Concepts of Hydrogels: Synthesis, Properties, and Their Applications. Polymers.

[177] Hu X., Jin M., Sun K., Zhang Z., Wu Z., Shi J., Liu P., Yao H., Wang D.A. (2024). Type II collagen scaffolds repair critical-sized osteochondral defects under induced conditions of osteoarthritis in rat knee joints via inhibiting TGF-beta-Smad1/5/8 signaling pathway. Bioact. Mater..

[178] Hernandez-Gonzalez A.C., Tellez-Jurado L., Rodriguez-Lorenzo L.M. (2020). Alginate hydrogels for bone tissue engineering, from injectables to bioprinting: A review. Carbohydr. Polym..

[179] Che X., Zhao T., Hu J., Yang K., Ma N., Li A., Sun Q., Ding C., Ding Q. (2024). Application of Chitosan-Based Hydrogel in Promoting Wound Healing: A Review. Polymers.

[180] Oprita E.I., Iosageanu A., Craciunescu O. (2023). Natural Polymeric Hydrogels Encapsulating Small Molecules for Diabetic Wound Healing. Gels.

[181] Bhattacharjee P., Ahearne M. (2022). Silk fibroin based interpenetrating network hydrogel for corneal stromal regeneration. Int. J. Biol. Macromol..

[182] Zhao L., Zhou Y., Zhang J., Liang H., Chen X., Tan H. (2023). Natural Polymer-Based Hydrogels: From Polymer to Biomedical Applications. Pharmaceutics.

[183] Sun L., Xu Y., Han Y., Cui J., Jing Z., Li D., Liu J., Xiao C., Li D., Cai B. (2023). Collagen-Based Hydrogels for Cartilage Regeneration. Orthop. Surg..

[184] Ahmadian E., Eftekhari A., Dizaj S.M., Sharifi S., Mokhtarpour M., Nasibova A.N., Khalilov R., Samiei M. (2019). The effect of hyaluronic acid hydrogels on dental pulp stem cells behavior. Int. J. Biol. Macromol..

[185] Wang S., Tavakoli S., Parvathaneni R.P., Nawale G.N., Oommen O.P., Hilborn J., Varghese O.P. (2022). Dynamic covalent crosslinked hyaluronic acid hydrogels and nanomaterials for biomedical applications. Biomater. Sci..

[186] Vijayavenkataraman S., Vialli N., Fuh J.Y.H., Lu W.F. (2019). Conductive collagen/polypyrrole-b-polycaprolactone hydrogel for bioprinting of neural tissue constructs. Int. J. Bioprint.

[187] Bonelli N., Poggi G., Chelazzi D., Giorgi R., Baglioni P. (2019). Poly(vinyl alcohol)/poly(vinyl pyrrolidone) hydrogels for the cleaning of art. J. Colloid Interface Sci..

[188] Munim S.A., Raza Z.A. (2018). Poly(lactic acid) based hydrogels: Formation, characteristics and biomedical applications. J. Porous Mater..

[189] Atta S., Khaliq S., Islam A., Javeria I., Jamil T., Athar M.M., Shafiq M.I., Ghaffar A. (2015). Injectable biopolymer based hydrogels for drug delivery applications. Int. J. Biol. Macromol..

[190] Rodríguez-Rodríguez R., García-Carvajal Z.Y., Jiménez-Palomar I., Jiménez-Avalos J.A., Espinosa-Andrews H. (2018). Development of gelatin/chitosan/PVA hydrogels: Thermal stability, water state, viscoelasticity, and cytotoxicity assays. J. Appl. Polym. Sci..

[191] Berkovitch Y., Seliktar D. (2017). Semi-synthetic hydrogel composition and stiffness regulate neuronal morphogenesis. Int. J. Pharm..

[192] Bustamante-Torres M., Romero-Fierro D., Arcentales-Vera B., Palomino K., Magana H., Bucio E. (2021). Hydrogels Classification According to the Physical or Chemical Interactions and as Stimuli-Sensitive Materials. Gels.

[193] Suhail M., Fang C.W., Chiu I.H., Hung M.C., Vu Q.L., Lin I.L., Wu P.C. (2022). Designing and In Vitro Characterization of pH-Sensitive Aspartic Acid-Graft-Poly(Acrylic Acid) Hydrogels as Controlled Drug Carriers. Gels.

[194] Gohy J.-F., Varshney S.K., Antoun S., Jérôme R. (2000). Water-Soluble Complexes Formed by Sodium Poly(4-styrenesulfonate) and a Poly(2-vinylpyridinium)-block-poly(ethyleneoxide) Copolymer. Macromolecules.

[195] Baker J., Stephens D., Blanch H., Prausnitz J. (1991). Swelling Equilibria for Acrylamide-Based Polyampholyte Hydrogels. Macromolecules.

[196] Casolaro M., Bottari S., Ito Y. (2006). Vinyl polymers based on L-histidine residues. Part 2. Swelling and electric behavior of smart poly(ampholyte) hydrogels for biomedical applications. Biomacromolecules.

[197] Alaghawani N.A., Alkhatib H., Elmancy L., Daou A. (2024). Harmonizing Innovations: An In-Depth Comparative Review on the Formulation, Applications, and Future Perspectives of Aerogels and Hydrogels in Pharmaceutical Sciences. Gels.

[198] Fan R., Cheng Y., Wang R., Zhang T., Zhang H., Li J., Song S., Zheng A. (2022). Thermosensitive Hydrogels and Advances in Their Application in Disease Therapy. Polymers.

[199] Chen X., Wang M., Yang X., Wang Y., Yu L., Sun J., Ding J. (2019). Injectable hydrogels for the sustained delivery of a HER2-targeted antibody for preventing local relapse of HER2+ breast cancer after breast-conserving surgery. Theranostics.

[200] Lachenmeier D.W. (2008). Safety evaluation of topical applications of ethanol on the skin and inside the oral cavity. J. Occup. Med. Toxicol..

[201] Zhang J.T., Huang S.W., Zhuo R.X. (2004). Temperature-sensitive polyamidoamine dendrimer/poly(N-isopropylacrylamide) hydrogels with improved responsive properties. Macromol. Biosci..

[202] Lu L., Wang T., Fang C., Song L., Qian C., Lv Z., Fang Y., Liu X., Yu X., Xu X. (2022). Oncolytic Impediment/Promotion Balance Disruption by Sonosensitizer-Free Nanoplatforms Unfreezes Autophagy-Induced Resistance to Sonocatalytic Therapy. ACS Appl. Mater. Interfaces.

[203] Kumbhar P.R., Desai H., Desai V.M., Priya S., Rana V., Singhvi G. (2025). Versatility of emulgel in topical drug delivery transforming its expedition from bench to bedside. Expert Opin. Drug Deliv..

[204] Charyulu N.R., Joshi P., Dubey A., Shetty A. (2021). Emulgel: A Boon for Enhanced Topical Drug Delivery. J. Young Pharm..

[205] Milutinov J., Krstonosic V., Cirin D., Pavlovic N. (2023). Emulgels: Promising Carrier Systems for Food Ingredients and Drugs. Polymers.

[206] Zoller K., To D., Bernkop-Schnurch A. (2025). Biomedical applications of functional hydrogels: Innovative developments, relevant clinical trials and advanced products. Biomaterials.

[207] Olteanu G., Neacsu S.M., Joita F.A., Musuc A.M., Lupu E.C., Ionita-Mindrican C.B., Lupuliasa D., Mititelu M. (2024). Advancements in Regenerative Hydrogels in Skin Wound Treatment: A Comprehensive Review. Int. J. Mol. Sci..

[208] Almoshari Y. (2022). Novel Hydrogels for Topical Applications: An Updated Comprehensive Review Based on Source. Gels.

[209] Barnes T.M., Mijaljica D., Townley J.P., Spada F., Harrison I.P. (2021). Vehicles for Drug Delivery and Cosmetic Moisturizers: Review and Comparison. Pharmaceutics.

[210] Richard M.A., Paul C., Nijsten T., Gisondi P., Salavastru C., Taieb C., Trakatelli M., Puig L., Stratigos A. (2022). Prevalence of most common skin diseases in Europe: A population-based study. J. Eur. Acad. Dermatol. Venereol..

[211] Raina N., Pahwa R., Bhattacharya J., Paul A.K., Nissapatorn V., de Lourdes Pereira M., Oliveira S.M.R., Dolma K.G., Rahmatullah M., Wilairatana P. (2022). Drug Delivery Strategies and Biomedical Significance of Hydrogels: Translational Considerations. Pharmaceutics.

[212] Robert B., Warfield I., Louis F., Stumpf Westfield N.J. (1955). Acrylic Acid Polymer Laxative Compositions. Patent Application.

[213] Gabriel D., Mugnier T., Courthion H., Kranidioti K., Karagianni N., Denis M.C., Lapteva M., Kalia Y., Moller M., Gurny R. (2016). Improved topical delivery of tacrolimus: A novel composite hydrogel formulation for the treatment of psoriasis. J. Control. Release.

[214] Stein Gold L., Weiss J., Rueda M.J., Liu H., Tanghetti E. (2016). Moderate and Severe Inflammatory Acne Vulgaris Effectively Treated with Single-Agent Therapy by a New Fixed-Dose Combination Adapalene 0.3%/Benzoyl Peroxide 2.5% Gel: A Randomized, Double-Blind, Parallel-Group, Controlled Study. Am. J. Clin. Dermatol..

[215] Palakkal S., Cortial A., Frusic-Zlotkin M., Soroka Y., Tzur T., Nassar T., Benita S. (2023). Effect of cyclosporine A—Tempol topical gel for the treatment of alopecia and anti-inflammatory disorders. Int. J. Pharm..

[216] Gordon R. (2013). Skin cancer: An overview of epidemiology and risk factors. Semin. Oncol. Nurs..

[217] Hasan N., Nadaf A., Imran M., Jiba U., Sheikh A., Almalki W.H., Almujri S.S., Mohammed Y.H., Kesharwani P., Ahmad F.J. (2023). Skin cancer: Understanding the journey of transformation from conventional to advanced treatment approaches. Mol. Cancer.

[218] Swetter S.M., Tsao H., Bichakjian C.K., Curiel-Lewandrowski C., Elder D.E., Gershenwald J.E., Guild V., Grant-Kels J.M., Halpern A.C., Johnson T.M. (2019). Guidelines of care for the management of primary cutaneous melanoma. J. Am. Acad. Dermatol..

[219] Schmults C.D., Blitzblau R., Aasi S.Z., Alam M., Amini A., Bibee K., Bordeaux J., Chen P.L., Contreras C.M., DiMaio D. (2023). Basal Cell Skin Cancer, Version 2.2024, NCCN Clinical Practice Guidelines in Oncology. J. Natl. Compr. Canc. Netw..

[220] Garcia-Mouronte E., Berna-Rico E., de Nicolas-Ruanes B., Azcarraga-Llobet C., Alonso-Martinez de Salinas L., Bea-Ardebol S. (2023). Imiquimod as Local Immunotherapy in the Management of Premalignant Cutaneous Conditions and Skin Cancer. Int. J. Mol. Sci..

[221] Zhang X., Xie Y., Wang L. (2023). Rare Cutaneous Side Effects of Imiquimod: A Review on Its Mechanisms, Diagnosis, and Management. Dermatol. Ther. Heidelb..

[222] Werbel T., Cohen P.R. (2018). Topical Application of 5-Fluorouracil Associated with Distant Seborrheic Dermatitis-like Eruption: Case Report and Review of Seborrheic Dermatitis Cutaneous Reactions after Systemic or Topical Treatment with 5-Fluorouracil. Dermatol. Ther. Heidelb..

[223] Saraiva M.I.R., Portocarrero L.K.L., Vieira M., Swiczar B.C.C., Westin A.T. (2018). Ingenol mebutate in the treatment of actinic keratoses: Clearance rate and adverse effects. An. Bras. Dermatol..

[224] Pansuriya R., Doutch J., Parmar B., Kailasa S.K., Mahmoudi N., Hoskins C., Malek N.I. (2024). A bio-ionic liquid based self-healable and adhesive ionic hydrogel for the on-demand transdermal delivery of a chemotherapeutic drug. J. Mater. Chem. B.

[225] Nawaz A., Ullah S., Alnuwaiser M.A., Rehman F.U., Selim S., Al Jaouni S.K., Farid A. (2022). Formulation and Evaluation of Chitosan-Gelatin Thermosensitive Hydrogels Containing 5FU-Alginate Nanoparticles for Skin Delivery. Gels.

[226] Blohm-Mangone K., Burkett N.B., Tahsin S., Myrdal P.B., Aodah A., Ho B., Janda J., McComas M., Saboda K., Roe D.J. (2018). Pharmacological TLR4 Antagonism Using Topical Resatorvid Blocks Solar UV-Induced Skin Tumorigenesis in SKH-1 Mice. Cancer Prev. Res..

[227] Ruiz V.H., Encinas-Basurto D., Sun B., Eedara B.B., Dickinson S.E., Wondrak G.T., Chow H.S., Curiel-Lewandrowski C., Mansour H.M. (2022). Design, Physicochemical Characterization, and In Vitro Permeation of Innovative Resatorvid Topical Formulations for Targeted Skin Drug Delivery. Pharmaceutics.

[228] Bayoumi S.A., Dawaba A.M., Mansour A., Zalat Z.A., Ammar A.A. (2022). Ectoine gel transdermal formulation as a novel therapeutic approach in melanoma using 3D printed microneedles. Pharm. Dev. Technol..

[229] Mishra M., Barkat M.A., Misra C., Alanezi A.A., Ali A., Chaurawal N., Ali A., Preet S., Barkat H., Raza K. (2024). Lipid-based microemulsion gel for the topical delivery of methotrexate: An optimized, rheologically acceptable formulation with conducive dermatokinetics. Arch. Dermatol. Res..

[230] Zhang W., Qian S., Chen J., Jian T., Wang X., Zhu X., Dong Y., Fan G. (2024). Photo-Crosslinked Pro-Angiogenic Hydrogel Dressing for Wound Healing. Int. J. Mol. Sci..

[231] Gocmen G., Gonul O., Oktay N.S., Yarat A., Goker K. (2015). The antioxidant and anti-inflammatory efficiency of hyaluronic acid after third molar extraction. J. Craniomaxillofac. Surg..

[232] Moran H.B.T., Turley J.L., Andersson M., Lavelle E.C. (2018). Immunomodulatory properties of chitosan polymers. Biomaterials.

[233] Larouche J., Sheoran S., Maruyama K., Martino M.M. (2018). Immune Regulation of Skin Wound Healing: Mechanisms and Novel Therapeutic Targets. Adv. Wound Care.

[234] Liu S., Wei L., Huang J., Luo J., Weng Y., Chen J. (2025). Chitosan/Alginate-Based Hydrogel Loaded With VE-Cadherin/FGF as Scaffolds for Wound Repair in Different Degrees of Skin Burns. J. Biomed. Mater. Res. B Appl. Biomater..

[235] Esposito S., De Simone G., Pan A., Brambilla P., Gattuso G., Mastroianni C., Kertusha B., Contini C., Massoli L., Francisci D. (2019). Epidemiology and Microbiology of Skin and Soft Tissue Infections: Preliminary Results of a National Registry. J. Chemother..

[236] Grossi A.P., Ruggieri A., Vecchio A.D., Comandini A., Corio L., Calisti F., Loreto G.D., Almirante B. (2022). Skin infections in Europe: A retrospective study of incidence, patient characteristics and practice patterns. Int. J. Antimicrob. Agents.

[237] Furnica D.T., Dittmer S., Scharmann U., Meis J.F., Steinmann J., Rath P.M., Kirchhoff L. (2023). In Vitro and In Vivo Effect of the Imidazole Luliconazole against *Lomentospora prolificans* and *Scedosporium* spp. Microbiol. Spectr..

[238] Kaur M., Singh G., Shivgotra R., Singh M., Thakur S., Jain S.K. (2024). Prolonged Skin Retention of Luliconazole from SLNs Based Topical Gel Formulation Contributing to Ameliorated Antifungal Activity. AAPS PharmSciTech.

[239] Cross E.R., Coulter S.M., Pentlavalli S., Laverty G. (2021). Unravelling the antimicrobial activity of peptide hydrogel systems: Current and future perspectives. Soft Matter.

[240] Albadr A.A., Coulter S.M., Porter S.L., Thakur R.R.S., Laverty G. (2018). Ultrashort Self-Assembling Peptide Hydrogel for the Treatment of Fungal Infections. Gels.

[241] Chiloeches A., Zagora J., Placha D., Torres M.D.T., de la Fuente-Nunez C., Lopez-Fabal F., Gil-Romero Y., Fernandez-Garcia R., Fernandez-Garcia M., Echeverria C. (2023). Synergistic Combination of Antimicrobial Peptides and Cationic Polyitaconates in Multifunctional PLA Fibers. ACS Appl. Bio Mater..

[242] Lombardo D., Kiselev M.A. (2022). Methods of Liposomes Preparation: Formation and Control Factors of Versatile Nanocarriers for Biomedical and Nanomedicine Application. Pharmaceutics.

[243] Alshaer W., Nsairat H., Lafi Z., Hourani O.M., Al-Kadash A., Esawi E., Alkilany A.M. (2022). Quality by Design Approach in Liposomal Formulations: Robust Product Development. Molecules.

[244] Kant Shashi K.S. (2012). A complete review on: Liposomes. Int. Res. J. Pharm..

[245] Sharma A. (1997). Liposomes in drug delivery: Progress and limitations. Int. J. Pharm..

[246] Bozo T., Meszaros T., Mihaly J., Bota A., Kellermayer M.S.Z., Szebeni J., Kalman B. (2016). Aggregation of PEGylated liposomes driven by hydrophobic forces. Colloids Surf. B Biointerfaces.

[247] Eugster R., Orsi M., Buttitta G., Serafini N., Tiboni M., Casettari L., Reymond J.L., Aleandri S., Luciani P. (2024). Leveraging machine learning to streamline the development of liposomal drug delivery systems. J. Control. Release.

[248] Wang Y., Grainger D.W. (2022). Regulatory Considerations Specific to Liposome Drug Development as Complex Drug Products. Front. Drug Deliv..

[249] Gaspar R.S., Silva-Lima B., Magro F., Alcobia A., da Costa F.L., Feio J. (2020). Non-biological Complex Drugs (NBCDs): Complex Pharmaceuticals in Need of Individual Robust Clinical Assessment Before Any Therapeutic Equivalence Decision. Front. Med..

[250] Klein K., Stolk P., De Bruin M.L., Leufkens H.G.M., Crommelin D.J.A., De Vlieger J.S.B. (2019). The EU regulatory landscape of non-biological complex drugs (NBCDs) follow-on products: Observations and recommendations. Eur. J. Pharm. Sci..

[251] Lanigan R.S., Yamarik T.A. (2002). Final report on the safety assessment of BHT(1). Int. J. Toxicol..

[252] Atre P., Rizvi S.A.A. (2024). A brief overview of quality by design approach for developing pharmaceutical liposomes as nano-sized parenteral drug delivery systems. RSC Pharm..

[253] Agrawal S.S., Baliga V., Londhe V.Y. (2024). Liposomal Formulations: A Recent Update. Pharmaceutics.

[254] Peralta M.F., Guzman M.L., Perez A.P., Apezteguia G.A., Formica M.L., Romero E.L., Olivera M.E., Carrer D.C. (2018). Liposomes can both enhance or reduce drugs penetration through the skin. Sci. Rep..

[255] Hoare T.R., Kohane D.S. (2008). Hydrogels in drug delivery: Progress and challenges. Polymer.

[256] Kollerup Madsen B., Hilscher M., Zetner D., Rosenberg J. (2018). Adverse reactions of dimethyl sulfoxide in humans: A systematic review. F1000Research.

[257] Gopinathan U., Stapleton F., Sharma S., Willcox M.D., Sweeney D.F., Rao G.N., Holden B.A. (1997). Microbial contamination of hydrogel contact lenses. J. Appl. Microbiol..

[258] Ming Z., Han L., Bao M., Zhu H., Qiang S., Xue S., Liu W. (2021). Living Bacterial Hydrogels for Accelerated Infected Wound Healing. Adv. Sci..

[259] Dantas M.G., Reis S.A., Damasceno C.M., Rolim L.A., Rolim-Neto P.J., Carvalho F.O., Quintans-Junior L.J., Almeida J.R. (2016). Development and Evaluation of Stability of a Gel Formulation Containing the Monoterpene Borneol. Sci. World J..

[260] Das S., Wong A.B.H. (2020). Stabilization of ferulic acid in topical gel formulation via nanoencapsulation and pH optimization. Sci. Rep..

[261] Khadivi Y., Shakeri S., Arjmandmazidi S., Shokri J., Monajjemzadeh F. (2024). The effect of emulgel preparation on the stability of Kojic acid in the topical anti-hyperpigmentation products. J. Cosmet. Dermatol..

[262] European Medicines Agency (2003). ICH Topic Q 1 A (R2) Stability Testing of New Drug Substances and Products. https://www.ema.europa.eu/en/documents/scientific-guideline/ich-q-1-r2-stability-testing-new-drug-substances-and-products-step-5_en.pdf.

[263] U.S. Food and Drug Administration (Center for Drug Evaluation and Research.) (2022). In Vitro Permeation Test Studies for Topical Drug Products Submitted in ANDAs.

[264] Suman D., Office of Generic Drugs, Division of Bioequivalence (2017). In Vitro Bioequivalence Data for a Topical Product: Bioequivalence Review Perspective-Presentation Food and drug Administration on fda.gov Ocrober 20th.

[265] Han J.Y., La Fiandra J.N., DeVoe D.L. (2022). Microfluidic vortex focusing for high throughput synthesis of size-tunable liposomes. Nat. Commun..

[266] van Swaay D., deMello A. (2013). Microfluidic methods for forming liposomes. Lab Chip.

[267] Wang T., Yu T., Li W., Liu Q., Sung T.C., Higuchi A. (2024). Design and lyophilization of mRNA-encapsulating lipid nanoparticles. Int. J. Pharm..

[268] Massing U., Cicko S., Ziroli V. (2008). Dual asymmetric centrifugation (DAC)—A new technique for liposome preparation. J. Control. Release.

[269] Ingebrigtsen S.G., Skalko-Basnet N., de Albuquerque Cavalcanti Jacobsen C., Holsaeter A.M. (2017). Successful co-encapsulation of benzoyl peroxide and chloramphenicol in liposomes by a novel manufacturing method—Dual asymmetric centrifugation. Eur. J. Pharm. Sci..

[270] Attri N., Das S., Banerjee J., Shamsuddin S.H., Dash S.K., Pramanik A. (2024). Liposomes to Cubosomes: The Evolution of Lipidic Nanocarriers and Their Cutting-Edge Biomedical Applications. ACS Appl. Bio Mater..

[271] Palma A.S., Casadei B.R., Lotierzo M.C., de Castro R.D., Barbosa L.R.S. (2023). A short review on the applicability and use of cubosomes as nanocarriers. Biophys. Rev..

[272] Sivadasan D., Sultan M.H., Alqahtani S.S., Javed S. (2023). Cubosomes in Drug Delivery-A Comprehensive Review on Its Structural Components, Preparation Techniques and Therapeutic Applications. Biomedicines.

[273] Globe Newswire-2019.-SunGen Pharma Receives Eighth ANDA Approval from US FDA. https://www.globenewswire.com/news-release/2019/10/31/1939048/0/en/SunGen-Pharma-Receives-Eighth-ANDA-Approval-from-US-FDA.html.

[274] Sacks S.L., Thisted R.A., Jones T.M., Barbarash R.A., Mikolich D.J., Ruoff G.E., Jorizzo J.L., Gunnill L.B., Katz D.H., Khalil M.H. (2001). Clinical efficacy of topical docosanol 10% cream for herpes simplex labialis: A multicenter, randomized, placebo-controlled trial. J. Am. Acad. Dermatol..

[275] O’Brien M.E., Wigler N., Inbar M., Rosso R., Grischke E., Santoro A., Catane R., Kieback D.G., Tomczak P., Ackland S.P. (2004). Reduced cardiotoxicity and comparable efficacy in a phase III trial of pegylated liposomal doxorubicin HCl (CAELYX/Doxil) versus conventional doxorubicin for first-line treatment of metastatic breast cancer. Ann. Oncol..

[276] Kontzias C., Zaino M., Feldman S.R. (2023). Tretinoin 0.1% and Benzoyl Peroxide 3% Cream for the Treatment of Facial Acne Vulgaris. Ann. Pharmacother..

[277] Bosslett M. FDA Approves Journey Medical’s DFD-29 for Rosacea. https://www.dermatologytimes.com/view/fda-approves-journey-medical-s-dfd-29-for-rosacea.

[278] Sousa R., Lakha D.R., Brette S., Hitier S. (2019). A Randomized, Double-Blind, Placebo-Controlled Study to Assess the Efficacy and Safety of Ambroxol Hard-Boiled Lozenges in Patients with Acute Pharyngitis. Pulm Ther..

[279] Wang X., Wang Z., Sun L., Liu H., Zhang F. (2022). Efficacy and safety of dapsone gel for acne: A systematic review and meta-analysis. Ann. Palliat. Med..

[280] Stotland M., Shalita A.R., Kissling R.F. (2009). Dapsone 5% gel: A review of its efficacy and safety in the treatment of acne vulgaris. Am. J. Clin. Dermatol..

[281] Mahmoudi C., Tahraoui Douma N., Mahmoudi H., Iurciuc Tincu C.E., Popa M. (2024). Hydrogels Based on Proteins Cross-Linked with Carbonyl Derivatives of Polysaccharides, with Biomedical Applications. Int. J. Mol. Sci..

[282] Liu M., Jin J., Zhong X., Liu L., Tang C., Cai L. (2024). Polysaccharide hydrogels for skin wound healing. Heliyon.

[283] Qin D., Cui Y., Zheng M., Yang Z., Wang X. (2025). Preparation of Ethosome Gel with Total Flavonoids from Vernonia anthelmintica (L.) Willd. for the Treatment of Vitiligo. Gels.

[284] Munoz-Perez E., Rubio-Retama J., Cusso L., Igartua M., Hernandez R.M., Santos-Vizcaino E. (2024). 3D-printed Laponite/Alginate hydrogel-based suppositories for versatile drug loading and release. Drug Deliv. Transl. Res..

[285] Protsak I.S., Morozov Y.M. (2025). Fundamentals and Advances in Stimuli-Responsive Hydrogels and Their Applications: A Review. Gels.

[286] Landolina J.A. (2020). In-Situ Cross-Linkable Polymeric Compositions and Methods Thereof.

[287] Landolina J.A., Ahmad O.M. (2016). Highly Efficacious Hemostatic Adhesive Polymer Scaffold.

[288] Shin B., Hillyer T., Shin W.S. (2024). Rational Design and Testing of Antibacterial Aloe Vera Hemostatic Hydrogel. Gels.

